# Predictive performance of the SOFA 2.0 score for in-hospital mortality in patients with heat stroke: a multicenter, data-driven subphenotype study

**DOI:** 10.3389/fmed.2026.1818390

**Published:** 2026-04-13

**Authors:** Haoming Luo, Yirui Zhu, Tongtong Wang, Weihua Li, Haiyang Guo, Chen Ting, Yuanyuan Ou, Mengshan Guan, Yifeng Zhang, Guoxuan Lin, Zhiguo Pan, Anwei Liu

**Affiliations:** 1 The First Clinical Medical School of Guangdong Pharmaceutical University, Guangzhou, China; 2 The Second Department of the Cadre Ward, General Hospital of Southern Theater Command, Guangzhou, China; 3 Department of Nursing Care, The General Hospital of Southern Theater Command, PLA, Guangzhou, China; 4 Department of Graduate School, Guangzhou University of Chinese Medicine, Guangzhou, Guangdong, China; 5 The First Clinical Medical College, Southern Medical University, Guangzhou, Guangdong, China; 6 Department of Hematology, General Hospital of Southern Theater Command, Guangzhou, China; 7 Department of Emergency Medicine, General Hospital of Southern Theater Command of PLA, Guangzhou, Guangdong, China; 8 War Trauma Treatment Center, The General Hospital of Southern Theater Command, PLA, Guangzhou, China

**Keywords:** critical illness, in-hospital mortality, machine learning, prognostic score, risk stratification

## Abstract

**Objective:**

Heat stroke can progress rapidly, and the risk of in-hospital mortality increases once multiple organ dysfunction develops. Early risk stratification is therefore clinically important, yet comparative evidence across commonly used severity scores in heat stroke remains limited.

**Methods:**

We conducted a multicenter retrospective cohort study of patients admitted with a first diagnosis of heat stroke to two tertiary hospitals in China between 2013 and 2023. The recalibrated SOFA 2.0 score (SOFA2), original SOFA, Modified Early Warning Score, National Early Warning Score, and Heat Stroke Severity Score were calculated using the first available data within 24 h of admission. In-hospital death was the primary outcome, with discharge alive treated as a competing event. Cumulative incidence functions and Fine–Gray models were used to assess risk gradients, and unsupervised clustering based on early clinical and laboratory features was applied to identify clinical subtypes.

**Results:**

Among 292 patients (mean age 29.8 ± 14.9 years), 24 (8.2%) died during hospitalization. The cumulative incidence of in-hospital death increased stepwise across SOFA2 quartiles (Gray test, *P* < 0.001), whereas separation across original SOFA quartiles was less distinct. Higher SOFA2 scores were associated with an increased risk of mortality risk, with spline analyses indicating a generally monotonic risk increase. Two major clinical subtypes were identified; in the higher-risk subtype identified by data-driven clustering, SOFA2 showed numerically consistent discrimination and stable net benefit trends; however, these subtype-specific findings should be interpreted cautiously.

**Conclusions:**

SOFA2 may provide an early, continuous representation of in-hospital mortality risk in patients with heat stroke, although external validation is required.

## 1 Introduction

Heat stroke is an acute and life-threatening condition caused by exposure to high environmental temperatures, with the potential for rapid progression to multiple organ dysfunction and a substantial risk of in-hospital mortality (1–4). Severe heat stroke (operational definition; corresponding to heat stroke with CNS dysfunction and/or organ dysfunction) is typically characterized by central nervous system dysfunction accompanied by systemic organ injury, and affected patients often require intensified monitoring and advanced critical care support (1–4). Accordingly, accurate early risk stratification at hospital admission is clinically important for guiding monitoring intensity and prioritizing healthcare resources.

In current clinical practice, commonly used severity scoring systems in critical care include the Sequential Organ Failure Assessment (SOFA) score, which focuses on multi-organ dysfunction, as well as vital sign–based early warning scores such as the Modified Early Warning Score (MEWS) and the National Early Warning Score (NEWS). In addition, a disease-specific scoring system for heat stroke, the Heat Stroke Severity Score (HSSS), has been proposed (6, 7, 9–13). However, these scoring systems were largely developed and validated in heterogeneous critically ill populations or infection-related cohorts. Whether their variable composition and risk gradients are appropriate for patients with heat stroke—an illness characterized by extreme heat stress and synchronous multi-organ injury—remains insufficiently evaluated in systematic comparative studies.

The recently proposed Sequential Organ Failure Assessment 2.0 score (SOFA2) recalibrates the thresholds of the original SOFA score (SOFA1) to achieve a more continuous relationship between score values and mortality risk, with the intent of better aligning risk estimates with contemporary ICU populations (8). To date, evidence supporting SOFA2 has been derived primarily from patients with infection-related critical illness or mixed ICU cohorts, and its predictive performance in non-infectious critical illness, particularly heat stroke and its severe forms, has not been adequately assessed.

Moreover, patients with heat stroke exhibit substantial heterogeneity in clinical presentation, disease progression, and outcome risk, and the performance of a given severity score may vary across different risk contexts. In recent years, unsupervised, data-driven phenotyping approaches have been increasingly applied in critical care to identify latent clinical subtypes with distinct physiological characteristics and outcome profiles without reliance on prespecified thresholds, and have been validated in populations with acute respiratory distress syndrome and sepsis (21, 22). Such approaches provide a structured framework for evaluating the applicability of severity scoring systems in the presence of real-world clinical heterogeneity.

On this basis, using a multicenter retrospective cohort from two tertiary hospitals, the present study systematically compared the performance of SOFA2 with several commonly used severity scoring systems in predicting in-hospital mortality among patients with heat stroke. By integrating data-driven phenotyping, we further evaluated the risk stratification performance of these scores across distinct clinical subtypes.

## 2 Materials and methods

### 2.1 Study design and participants

This study was a multicenter retrospective cohort study. Patients admitted with a diagnosis of heat stroke were screened from January 2013 to December 2023 at two tertiary hospitals in China.

Heat stroke was diagnosed according to international consensus criteria. Severe heat stroke was defined using an operational research definition as an acute heat-related illness accompanied by at least one of the following: (1) central nervous system dysfunction or altered mental status; and/or (2) organ dysfunction requiring close monitoring or supportive therapy (1–4, 6). Because reliable data on environmental exposure and prehospital core body temperature were unavailable in some cases, classification of severe heat stroke relied on the above operational criteria. Central nervous system dysfunction was defined as a Glasgow Coma Scale (GCS) score < 15 or a non-“Alert” status on the AVPU scale, consistent with previous observational studies (1–4, 6).

Patients were included if: (1) heat stroke–related illness was the primary reason for admission; and (2) relatively complete clinical data were available within 24 h of admission (< 10% missing items), allowing calculation of SOFA2 and other scoring systems. Exclusion criteria were: (1) hyperthermia or altered mental status attributable to non–heat-related etiologies (e.g., central nervous system infection, acute stroke, drug- or anesthesia-related hyperthermia); (2) pre-existing severe neurological disorders impairing assessment of consciousness; (3) repeated admissions during the same hospitalization; and (4) missing key variables or outcome information precluding score calculation or outcome ascertainment.

**Figure 1:**
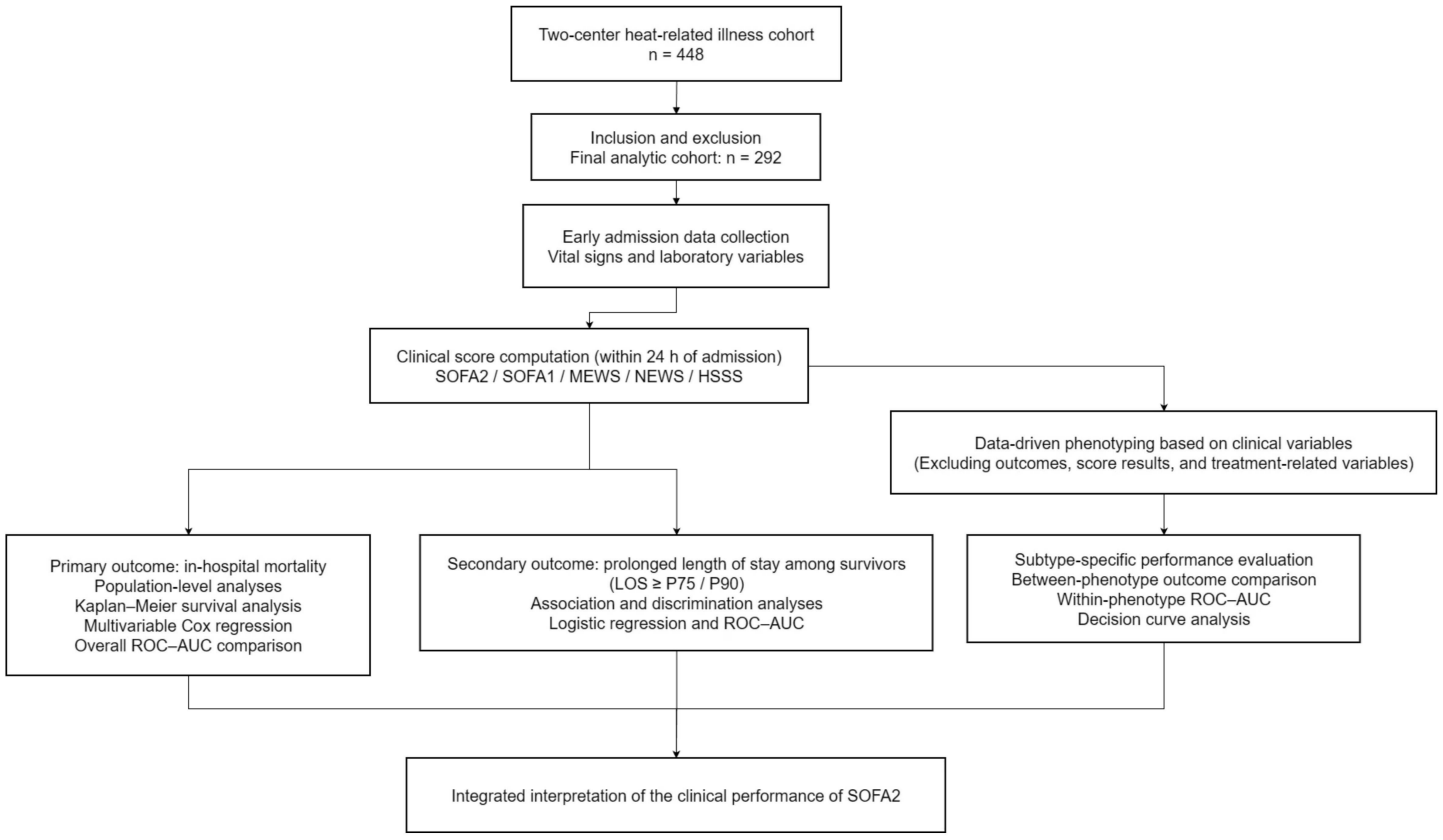
Schema of the study design and progress.

The General Research Process is summarized in Figure 1 and detailed in the Supplementary material (Figure 1). This study was reported in accordance with the STROBE statement (23), and relevant TRIPOD items were followed for analyses related to prediction model evaluation (24).

### 2.2 Data collection and variables

Demographic characteristics, vital signs, laboratory results, and selected treatment-related information were extracted from medical records. For each variable, the first available value within 24 h of hospital admission was used to represent baseline physiological status and to minimize the influence of subsequent interventions.

The original dataset consisted of 51 clinical variables, including neurological status, cardiopulmonary parameters, biochemical indices, and coagulation-related measures. Variables were used for severity score calculation and for descriptive analyses, outcome analyses, and unsupervised phenotyping. For phenotyping, only early physiological and laboratory variables were included, whereas outcomes, severity score results, and treatment- or organ support–related indicators (e.g., mechanical ventilation, vasoactive agents, and renal replacement therapy) were excluded to reduce information leakage.

### 2.3 Scoring systems and score calculation

For each patient, the following severity scoring systems were calculated according to their original definitions: SOFA1, SOFA2, MEWS, NEWS, and HSSS. SOFA2 was calculated strictly based on the most recent international consensus statement (8). SOFA1, MEWS, and NEWS were calculated according to their original publications or guideline-recommended methods (9, 11–13), and HSSS was calculated according to the Chinese expert consensus criteria for heat stroke (6). All scores were derived using the first available clinical data within 24 h of admission. No modifications were made to variable selection, weighting schemes, or cutoff thresholds.

For all five scoring systems (SOFA1, SOFA2, MEWS, NEWS, and HSSS), score calculation was available in the same 292 patients included in the analytic cohort.

For SOFA2, substitute scoring principles from the consensus document (8) were applied only when explicitly permitted by the official algorithm. In the present cohort, such substitution was required in only one patient: because the patient met the indication for renal replacement therapy (RRT) but did not actually undergo RRT, the renal component was assigned 4 points according to the official SOFA2 substitute scoring rule. No other SOFA2 component required substitute scoring.

The remaining scoring systems were calculated according to their original definitions without additional imputation.

### 2.4 Outcomes

The primary outcome was all-cause in-hospital mortality.

Among survivors, length-of-stay–related outcomes were defined as exploratory secondary endpoints. Prolonged hospitalization was defined as a hospital length of stay (LOS) at or above the 75th percentile (P75), and extremely prolonged hospitalization as an LOS at or above the 90th percentile (P90), based on the LOS distribution among survivors. LOS was defined as the number of days from admission to discharge or in-hospital death. Patients with LOS recorded as 0 days (typically reflecting hospitalization < 6 h or discharge against medical advice) were excluded from LOS-related analyses (see Statistical Analysis). Secondary analyses were restricted to survivors.

### 2.5 Statistical analysis

The overall analytic plan, including competing-risk regression, restricted cubic spline modeling, unsupervised clustering, and subtype-stratified discrimination analyses, was prespecified before formal outcome modeling. Given the limited number of in-hospital death events, particularly within subtype-stratified analyses, model complexity was kept intentionally low for regression models (typically score + age), and subtype-specific ROC and decision curve analyses were considered exploratory. To improve transparency regarding model complexity relative to the observed number of events, the approximate events-per-variable (EPV) or effective degrees of freedom for the main regression analyses are summarized in Supplementary Table 4.

#### 2.5.1 Baseline comparisons and survival analyses

For descriptive presentation of risk gradients, SOFA1 and SOFA2 were categorized into quartiles for primary analyses and dichotomized by median for supplementary display. For baseline comparisons, patients were divided into low and high SOFA2 groups using the cohort median SOFA2 score as the cutoff. To minimize circular reasoning, SOFA2 component variables and treatment- or organ support–related indicators were excluded. Between-group imbalance was quantified using absolute standardized mean differences (|SMD|), with |SMD| ≥ 0.2 considered potentially meaningful. Continuous variables were compared using the Wilcoxon rank-sum test.

**Figure 2:**
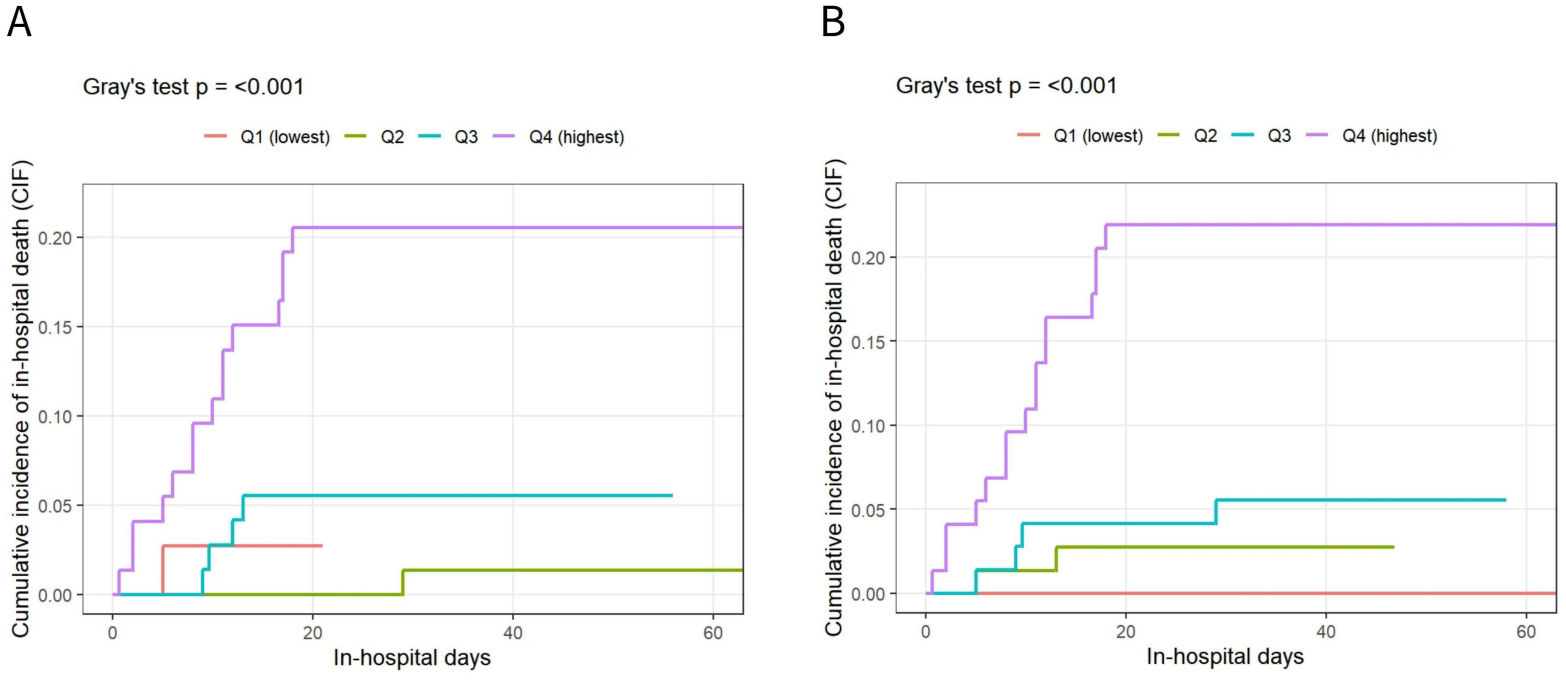
Cumulative incidence of in-hospital death across SOFA1 and SOFA2 quartiles. **(A)** Cumulative incidence curves of in-hospital mortality stratified by quartiles of the SOFA1 score. **(B)** Cumulative incidence curves of in-hospital mortality stratified by quartiles of the SOFA2 score. Discharge was treated as a competing event. The cumulative incidence function (CIF) was used to estimate the risk of in-hospital mortality, and differences among quartile groups were compared using Gray's test. The x-axis indicates hospital days, and the y-axis represents the cumulative incidence of in-hospital death. Curves are color-coded according to SOFA quartile groups (Q1–Q4). Curves are displayed up to hospital day 60 to avoid unstable estimates due to a small number of patients remaining at risk.

**Figure 3:**
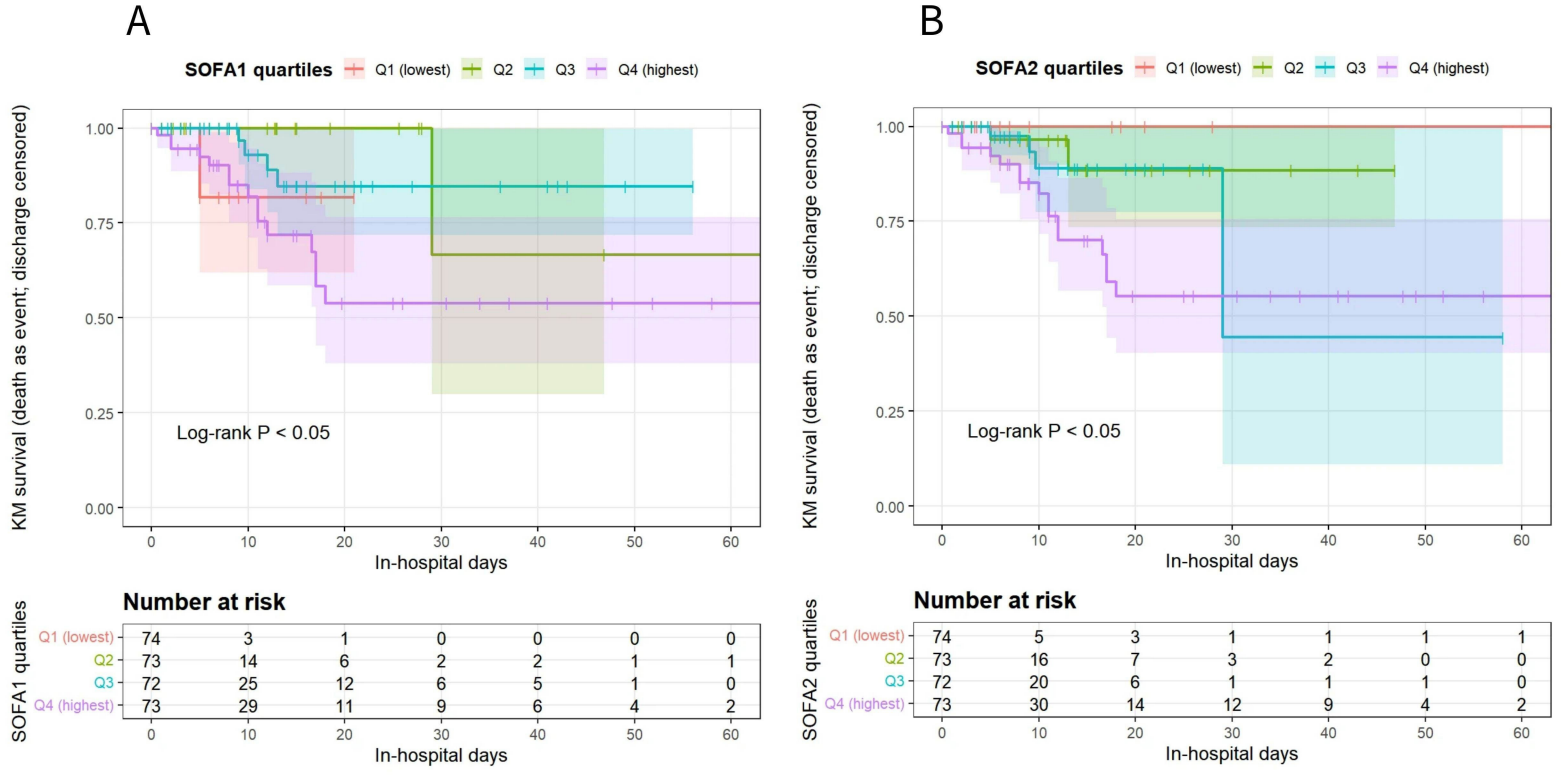
Kaplan–Meier survival curves across SOFA1 and SOFA2 quartiles. **(A)** Kaplan–Meier survival curves during hospitalization stratified by quartiles of the SOFA1 score. **(B)** Kaplan–Meier survival curves during hospitalization stratified by quartiles of the SOFA2 score. In the Kaplan–Meier analysis, in-hospital death was defined as the event, and discharge was treated as censoring. Survival curves were compared among quartile groups using the log-rank test. The x-axis indicates hospital days, and the y-axis represents the Kaplan–Meier estimated survival probability. Curves are color-coded according to SOFA quartile groups (Q1–Q4). Curves are displayed up to hospital day 60 to avoid unstable estimates due to a small number of patients remaining at risk. The number of patients at risk at each time point is shown below the plot.

Because discharge alive precludes in-hospital death and constitutes a competing event, primary time-to-event analyses were conducted using competing-risk methods. In-hospital death was treated as the event of interest and discharge alive as the competing event. Cumulative incidence functions (CIFs) were compared across SOFA quartile groups using Gray's test (Figure 2). Kaplan–Meier analyses were performed as supplementary visualization only, treating discharge alive as censoring and comparing groups using the log-rank test (Figure 3; Supplementary Figure 1). All curves were truncated at 60 days.

#### 2.5.2 Regression analyses of severity scores and in-hospital mortality

Associations between severity scores and in-hospital mortality were evaluated using Fine–Gray subdistribution hazard regression models, treating discharge alive as the competing event. Results were reported as subdistribution hazard ratios (sHRs) with 95% confidence intervals.

Each scoring system (SOFA2, SOFA1, MEWS, NEWS, and HSSS) was entered as a continuous predictor. Given the limited number of events, models were adjusted for age only. Restricted cubic splines (RCS) were incorporated to assess potential nonlinearity. Overall association was evaluated using the global test (P_overall), and deviation from linearity was assessed using the nonlinear component test (P_nonlinear). Sensitivity analyses were conducted using cause-specific Cox proportional hazards models (14), with proportional hazards assumptions assessed using Schoenfeld residuals (Supplementary Figure 5). These analyses were intended for prognostic association and risk stratification rather than causal inference.

#### 2.5.3 Data-driven patient phenotyping

To address heterogeneity in early clinical presentation, unsupervised phenotyping was performed. The clustering feature matrix included only physiological and laboratory variables obtained within 24 h of admission. Outcome variables, severity score results, and treatment- or organ support–related indicators (e.g., mechanical ventilation, vasoactive agents, and renal replacement therapy) were excluded. This design was intended to reduce direct information leakage from prognostic scores or downstream management decisions; however, because early physiological and laboratory abnormalities may still reflect overall illness severity, the resulting subtypes were interpreted as data-driven early clinical profiles rather than fully distinct biological endotypes.

Selected continuous variables were log-transformed when appropriate, and robust scaling was applied. Dimensionality reduction was performed using principal component analysis (PCA), with the number of retained components determined by cumulative explained variance (19). Clustering was conducted in PCA space using a Gaussian mixture model (GMM) (18). The optimal number of clusters was selected based on Bayesian information criterion (BIC) and bootstrap-derived stability metrics, including the adjusted Rand index (ARI) (20).

Variables with >50% missingness were excluded before clustering. Remaining missing values were handled using multivariate iterative imputation. To reduce distortion from extreme observations, a two-stage rare/outlier detection strategy was applied based on the tail distribution of individual negative log-likelihood (NLL) values from the GMM, using the 99th percentile as an initial threshold and constraining rare samples to approximately 3%. Nine individuals were classified as rare/outliers and analyzed separately. Primary analyses focused on the regular subtypes (Subtype 0 and Subtype 1). Additional details are provided in the Supplementary methods and Supplementary Figures 2, 3.

#### 2.5.4 Predictive performance and clinical decision analysis

Discriminative performance for in-hospital mortality was assessed using receiver operating characteristic (ROC) curves and area under the curve (AUC) in the overall cohort and within phenotypic subtypes (15).

Decision curve analysis (DCA) was conducted to compare net clinical benefit across a range of threshold probabilities. For each scoring system, a univariable logistic regression model was fitted with in-hospital mortality as the outcome and the score as a continuous predictor. Predicted probabilities were used to compute net benefit. Given the small number of deaths in Subtype 0, DCA was primarily performed in Subtype 1 using complete-case patients with complete score data available (*n* = 135 of 136), and compared with default “treat all” and “treat none” strategies. These analyses were intended to provide exploratory and descriptive comparisons of relative net benefit trends across scoring systems within the same cohort, rather than to establish definitive clinical decision thresholds. Model calibration was not evaluated as a primary endpoint and warrants dedicated assessment in independent external validation cohorts before clinical implementation (16, 20).

#### 2.5.5 Secondary analyses of prolonged length of hospital stay among survivors

Among survivors, patients with LOS recorded as 0 days were excluded. Prolonged LOS (≥P75) and extremely prolonged LOS (≥P90) were treated as binary outcomes.

Univariable logistic regression models were used to evaluate associations between admission severity scores (SOFA1, SOFA2, MEWS, NEWS, and HSSS) and LOS-related outcomes, reporting odds ratios (ORs) with 95% confidence intervals. Discriminative performance was assessed using ROC curves and AUC. These analyses were exploratory and supplementary.

## 3 Results

### 3.1 Baseline characteristics

A total of 292 patients with heat stroke–related diagnoses were included in the study. The mean age was 29.8 ± 14.9 years. Central nervous system dysfunction at admission was observed in 236 patients (80.9%). During hospitalization, 24 patients (8.2%) died, and 211 patients (72.3%) were admitted to the intensive care unit. Under the applicable ethics and confidentiality framework, the cohort did not include military-related personnel. Exertional heat stroke was identified in 27 patients (9.2%), while patients aged < 18 years and >50 years accounted for 10.3% and 13.3% of the cohort, respectively.

Based on the median SOFA2 score at admission (5 points), patients were classified into a low SOFA2 group (*n* = 162) and a high SOFA2 group (*n* = 130).

**Figure 4:**
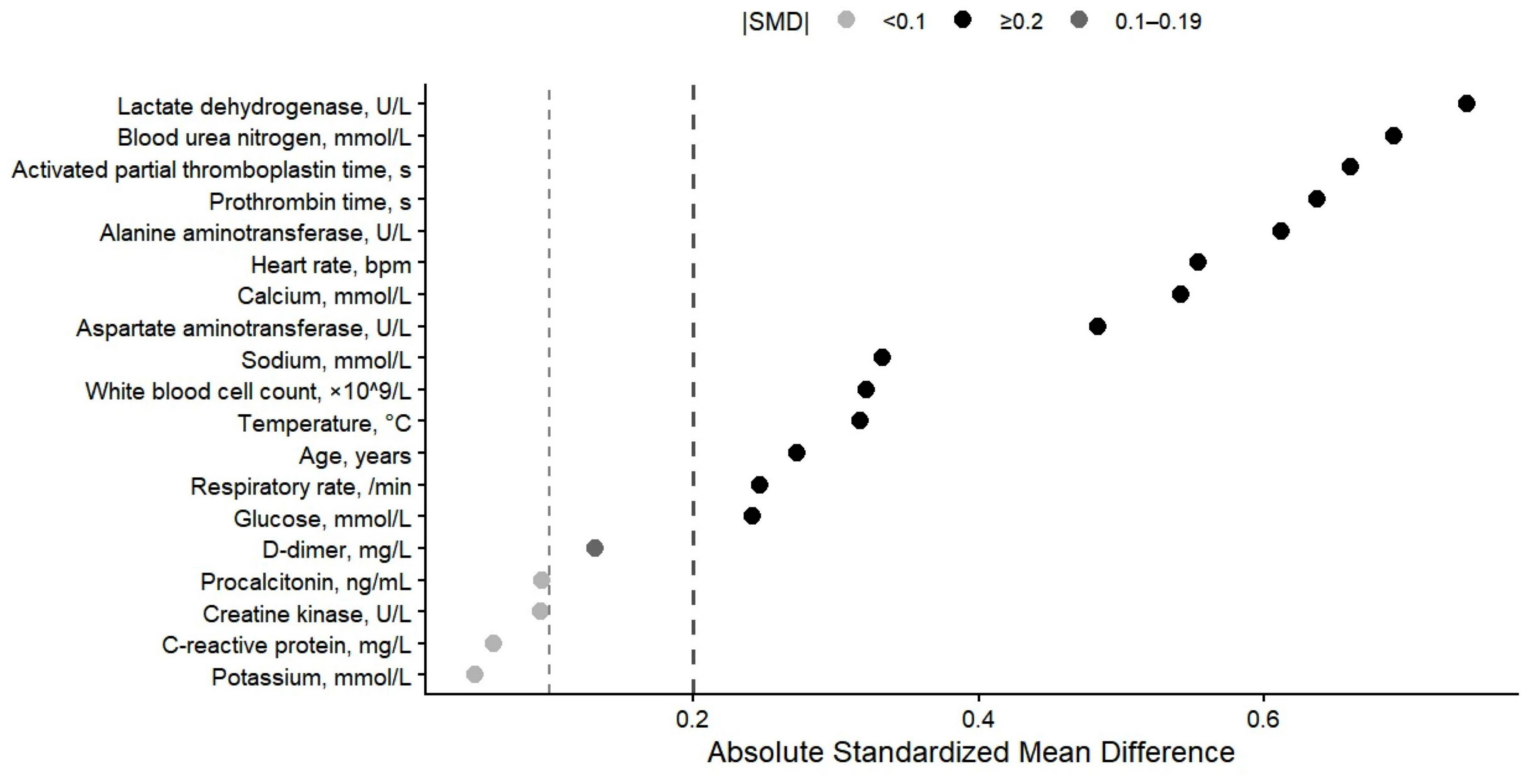
Baseline covariate imbalance between low and high SOFA2 groups. Baseline differences between the two groups were assessed using the absolute standardized mean difference (|SMD|). Each point represents the |SMD| value of a non-SOFA2 component variable between groups. Dashed lines indicate commonly used imbalance thresholds (|SMD| = 0.1 and 0.2), with |SMD| ≥ 0.2 generally considered potentially clinically meaningful. To avoid circular reasoning, none of the variables shown in this figure were included in the calculation of the SOFA2 score.

Baseline characteristics between the two groups were compared using the absolute standardized mean difference (|SMD|) to quantify imbalance in variables not included in the SOFA2 score (Figure 4). Specific values are provided in the Supplementary Table 1. Several variables reflecting metabolic derangement, organ dysfunction, and coagulation abnormalities showed moderate to large imbalance between the low and high SOFA2 groups (|SMD| ≥ 0.2).

Compared with the low SOFA2 group, patients in the high SOFA2 group more frequently exhibited abnormalities in markers related to organ injury and coagulation, along with differences in circulatory and liver function–related variables. In contrast, imbalance in selected inflammatory and electrolyte-related variables was relatively limited.

All five severity scores were successfully calculated in the same 292 patients. For SOFA2, substitute scoring was applied in only one patient according to the official consensus rule for a patient who met the indication for RRT but did not undergo RRT.

### 3.2 Association between SOFA scores and in-hospital survival outcomes

During hospitalization, a total of 24 all-cause deaths occurred (8.2%). To compare in-hospital mortality risk across different severity score strata while accounting for discharge alive as a competing event, competing-risk analyses were performed for SOFA1 and SOFA2.

Results of the competing-risk analysis stratified by SOFA1 quartiles are shown in Figure 2A (Figure 2). As SOFA1 score levels increased, the cumulative incidence of in-hospital death showed an upward trend, with statistically significant differences observed across groups (Gray's test, *P* < 0.001). Overall, SOFA1 demonstrated some ability to separate patients across different risk levels; however, separation between intermediate quartile groups was relatively limited.

Competing-risk analysis stratified by SOFA2 quartiles is presented in Figure 2B. With increasing SOFA2 quartiles, the cumulative incidence of in-hospital death exhibited a more clearly stepwise increase (Gray's test, *P* < 0.001), and separation between quartile groups was apparent early during hospitalization.

As summarized in Figure 2, compared with SOFA1, SOFA2 showed a more consistent gradient in risk stratification for in-hospital mortality, with clearer separation across severity strata.

To avoid instability related to small numbers at risk, cumulative incidence curves are displayed up to 60 days after hospital admission.

As supplementary descriptive analyses, Kaplan–Meier survival curves stratified by SOFA quartiles are shown in Figure 3A for SOFA1 and Figure 3B for SOFA2 (Figure 3). Overall trends were consistent with the competing-risk analyses. It should be noted that, in Kaplan–Meier analyses, discharge alive was treated as censoring and competing events were not explicitly accounted for; therefore, these results are presented for descriptive purposes only.

### 3.3 Multivariable analyses of SOFA scores and in-hospital mortality

Because discharge alive constitutes a competing event for in-hospital death, associations between SOFA scores and in-hospital mortality were further evaluated within a competing-risk framework. Fine–Gray subdistribution hazard regression models were applied, with age included as the sole adjustment variable under a parsimonious adjustment strategy, to estimate associations between SOFA scores and the cumulative incidence of in-hospital death.

**Figure 5:**
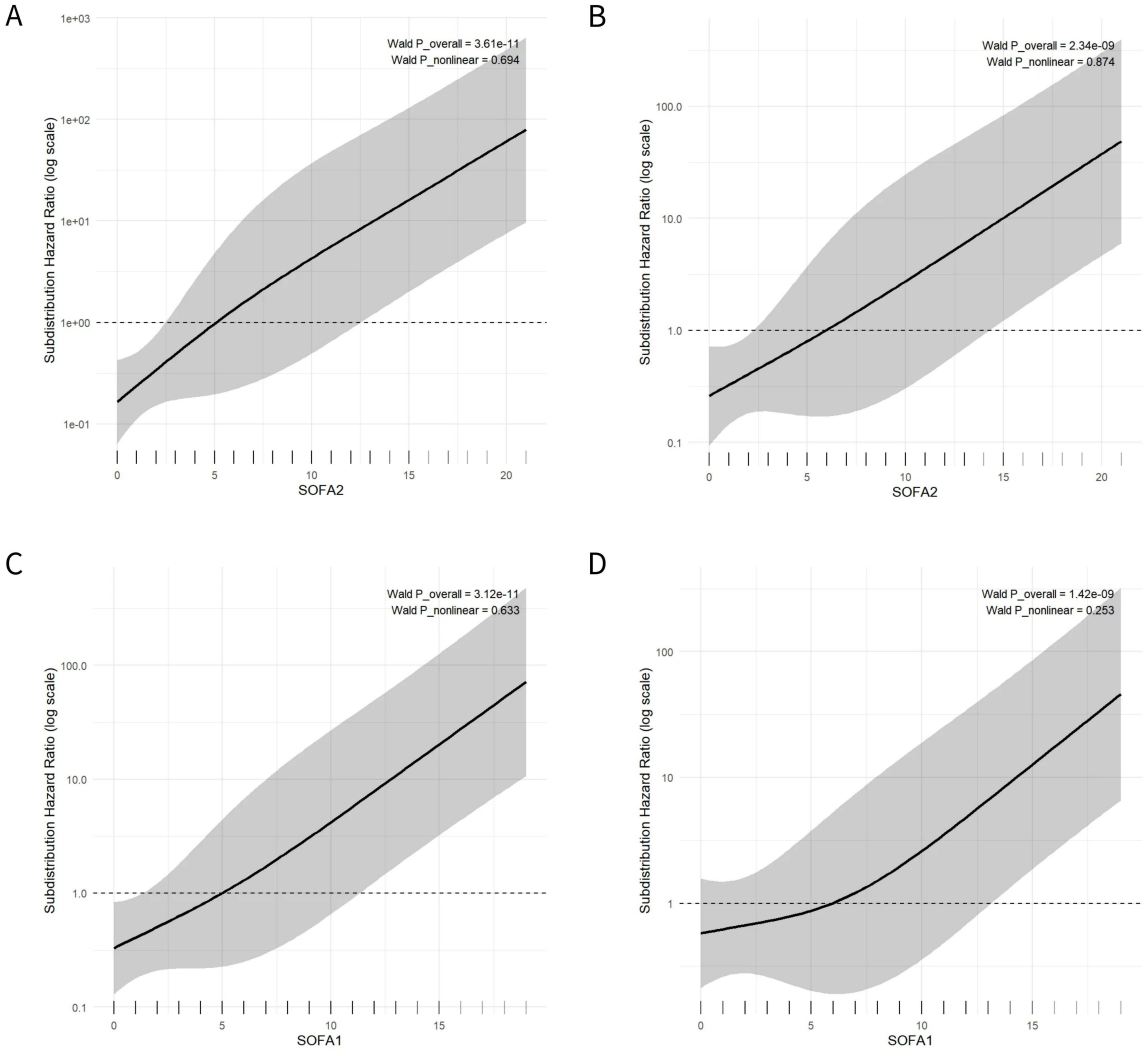
Restricted cubic spline (RCS) plots from multivariable Fine–Gray competing-risk models for the associations of SOFA2 and SOFA1 scores with in-hospital mortality. **(A, B)** Restricted cubic spline (RCS) curves from multivariable Fine–Gray (subdistribution hazard) models for the SOFA2 score in the overall cohort and the ICU-only cohort, respectively. **(C, D)** RCS curves from multivariable Fine–Gray models for the SOFA1 score in the overall cohort and the ICU-only cohort, respectively. The x-axis represents the SOFA score, and the y-axis indicates the subdistribution hazard ratio (sHR; log scale). The solid line shows the estimated risk trend, and the shaded area denotes the 95% confidence interval. The dashed line indicates the reference level (sHR = 1). Rug marks along the bottom of the x-axis represent the distribution of observed values. The annotated P_overall and P_nonlinear values (Wald test) assess the overall association and the presence of nonlinearity, respectively.

To characterize the dose–response pattern between SOFA scores treated as continuous variables and in-hospital mortality risk, restricted cubic splines (RCS) were incorporated into the Fine–Gray models. Overall associations were assessed using the global test (P_overall), and departures from linearity were evaluated using the test for the nonlinear component (P_nonlinear) (Figure 5).

In the overall cohort, SOFA2 showed a significant overall association with in-hospital mortality risk (P_overall = 3.61 × 10^−11^), with no evidence of nonlinearity (P_nonlinear = 0.694) (Figure 5A). Consistent results were observed in the ICU subgroup (P_overall = 2.34 × 10^−9^; P_nonlinear = 0.874) (Figure 5B). Across the observed range, increasing SOFA2 scores were associated with a monotonic increase in the cumulative incidence of in-hospital death, without statistically significant deviation from a linear trend.

For SOFA1, RCS analyses similarly demonstrated significant overall associations with in-hospital mortality. In the overall cohort, P_overall was 3.12 × 10^−11^ and P_nonlinear was 0.633 (Figure 5C), while in the ICU subgroup, P_overall was 1.42 × 10^−9^ and P_nonlinear was 0.253 (Figure 5D). For both scores, risk curves showed a progressive increase in mortality risk with increasing score values, and tests for nonlinearity did not indicate significant departures from linearity.

To assess the robustness of the primary findings, sensitivity analyses were further performed using cause-specific multivariable Cox proportional hazards models (Supplementary Figure 5), based on the results of the univariable Cox regression analyses (Supplementary Table 2). The overall direction and magnitude of the associations were consistent with those observed in the competing-risk regression analyses (Figure 5).

The main adjusted regression models were intentionally parsimonious (score + age). Across the overall-cohort regression analyses, the approximate EPV was 12 for linear models and 8 for restricted cubic spline models with 3 knots (Supplementary Table 4).

### 3.4 Unsupervised identification of patient subtypes

A total of 292 patients were included in the unsupervised clustering analysis. To minimize the risk of information leakage and circular reasoning, outcome variables, severity score results, and treatment- or organ support–related indicators were excluded from the feature matrix. Only physiological parameters and laboratory measurements first available within 24 h of hospital admission were included, resulting in a final set of 28 core variables for subtype identification.

**Figure 6:**
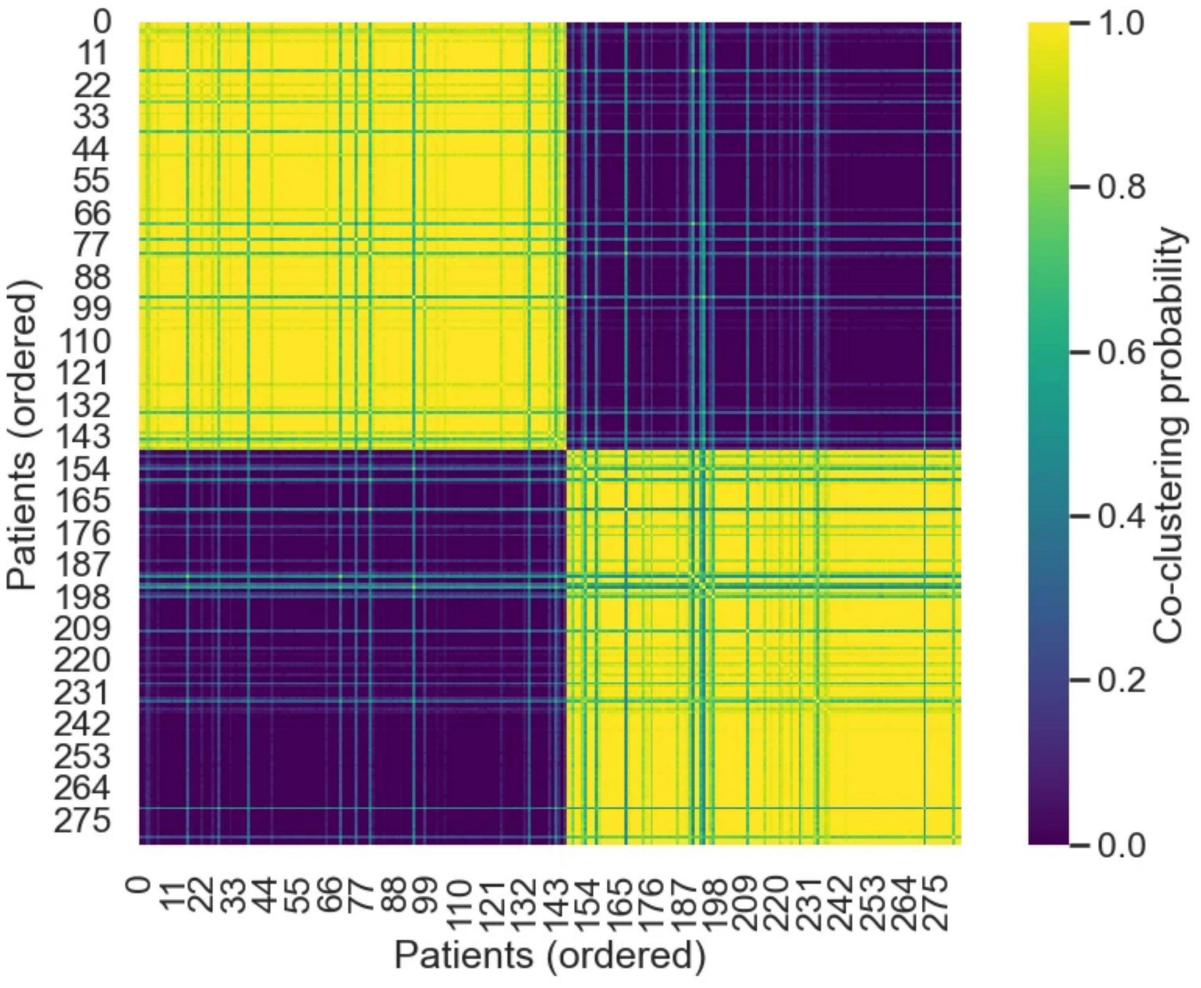
Consensus clustering matrix of the CONV population. This figure shows the consensus clustering matrix constructed during repeated subsampling-based clustering in the conventional cohort after exclusion of outlier samples (CONV, *n* = 283). Both rows and columns represent individual patients, ordered according to the final merged subtype assignments. Each matrix element indicates the probability (range, 0–1) that any two patients were assigned to the same cluster across multiple clustering iterations. Values closer to 1 reflect more stable co-clustering and consistent subtype membership across resampling runs. The matrix exhibits a clear block-diagonal pattern, suggesting good clustering consensus and robust subtype stability among the identified clinical subphenotypes.

Missing values were handled using multivariate iterative imputation, and all variables were subjected to robust scaling. Dimensionality reduction was then performed using principal component analysis (PCA), followed by clustering in the reduced feature space using a Gaussian mixture model (GMM). Selection of the optimal number of clusters (K) was based on a combination of the Bayesian information criterion (BIC) and cluster stability metrics derived from bootstrap resampling, specifically the adjusted Rand index (ARI). To further assess robustness, consensus clustering was applied across a range of cluster numbers (K = 2–6). The results indicated that K = 2 yielded the most stable clustering structure, with high within-cluster consistency and clear separation between clusters (Figure 6); therefore, K = 2 was selected for subsequent analyses.

To reduce the potential influence of extreme observations on cluster structure, nine rare/outlier individuals (3.1% of the total sample) were identified based on the tail distribution of individual negative log-likelihood (NLL) values from the GMM. These patients were excluded from construction of the main subtypes. The remaining 283 patients constituted the conventional (CONV) cohort and were included in the final clustering analysis.

Within the regular sample, patients were classified into two major clinical subtypes: Subtype 1 (*n* = 136) and Subtype 0 (*n* = 147). The consensus matrix exhibited a clear block-diagonal structure, indicating high within-cluster co-clustering probabilities and robust subtype stability across resampling iterations (Figure 6).

**Figure 7:**
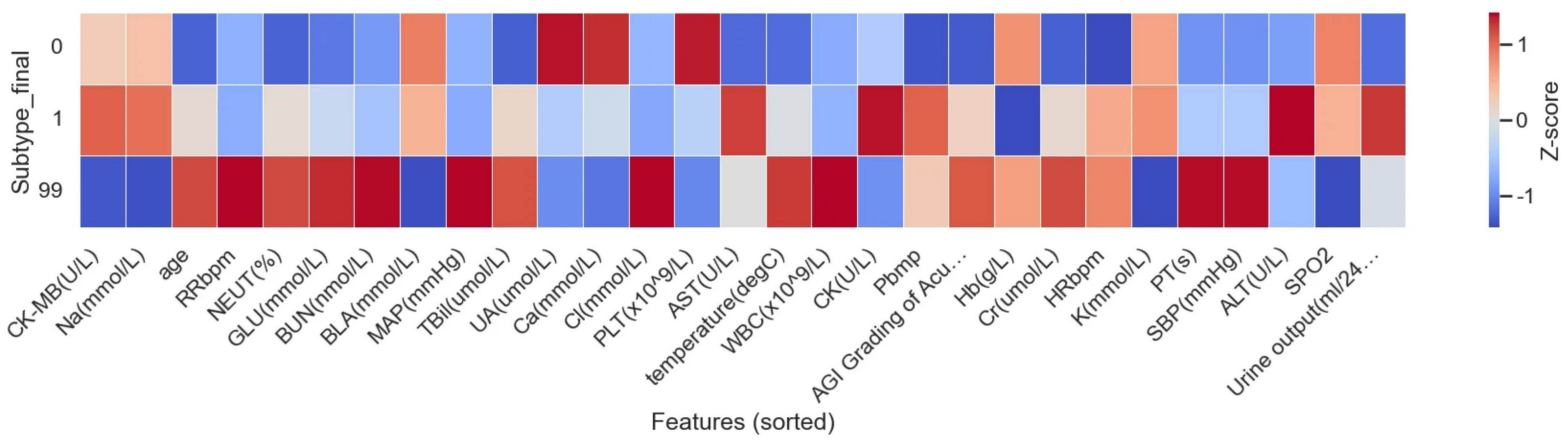
Z-Score heatmap of clinical features across patient subtypes. This heatmap illustrates differences in early admission clinical, physiological, and laboratory features across patient subgroups (Subtype 0, Subtype 1, and rare/outlier). Each column represents a clinical variable, and each row corresponds to a patient subtype. All variables were Z-score standardized, with colors ranging from blue to red indicating lower to higher values relative to the overall mean. The heatmap highlights distinct patterns of physiological status and organ dysfunction at admission among subtypes, providing a basis for phenotypic interpretation of the identified clinical subphenotypes.

The Z-score–standardized heatmap (Figure 7) showed that patients in Subtype 1 exhibited more pronounced abnormalities in organ function–related features, including higher heart rate, plasma BNP levels (Pbnp), age, and liver injury–related markers (ALT/AST), along with alterations in serum calcium levels and platelet counts. In contrast, patients in Subtype 0 displayed overall physiological and laboratory profiles closer to normal ranges.

In-hospital outcomes differed substantially between the two subtypes. The in-hospital mortality rate was 15.44% (21/136) in Subtype 1, compared with 2.04% (3/147) in Subtype 0. No deaths were observed in the rare/outlier subgroup (0/9); however, given the limited sample size, the clinical significance of this finding requires further evaluation.

### 3.5 Comparative performance of severity scores within clinical subtypes

**Figure 8:**
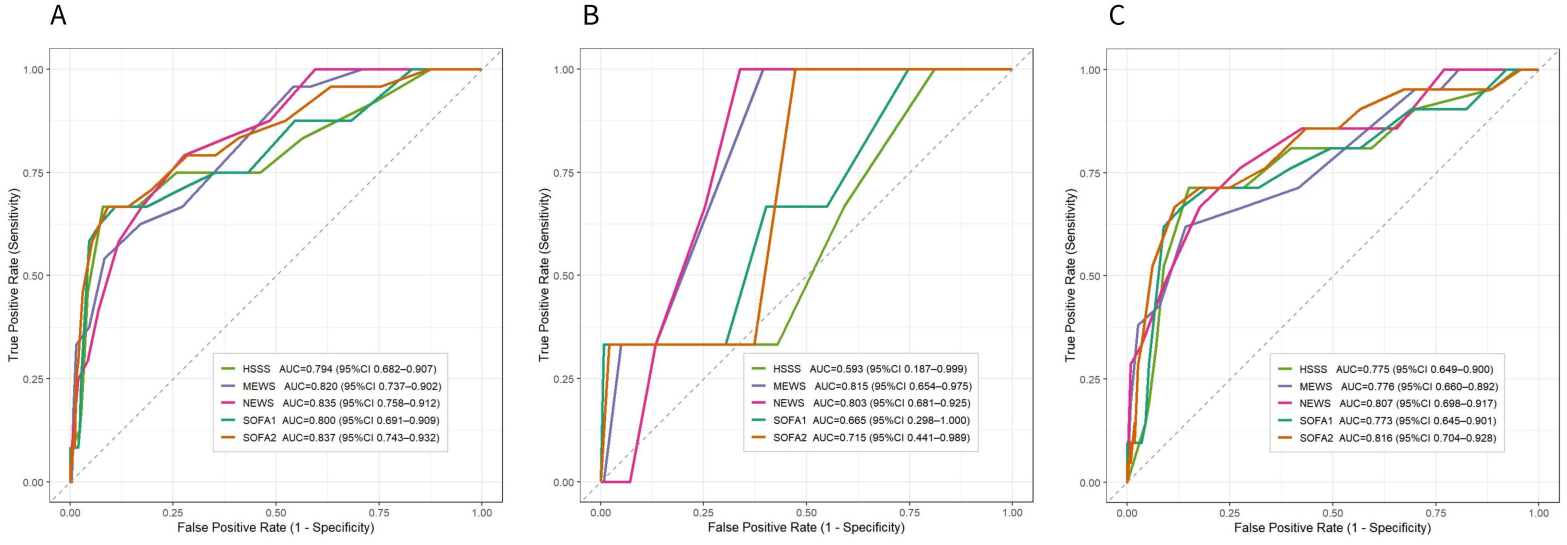
ROC curve analysis of different scoring systems for in-hospital mortality in the overall cohort and clinical subtypes. **(A)** Receiver operating characteristic (ROC) curves of HSSS, MEWS, NEWS, SOFA1, and SOFA2 in the overall cohort. **(B)** ROC curves of the corresponding scoring systems in Subtype 0. **(C)** ROC curves of the corresponding scoring systems in Subtype 1. The x-axis represents the false-positive rate (1 – specificity), and the y-axis indicates the true-positive rate (sensitivity). The dashed line denotes the reference line for random classification. The area under the curve (AUC) with 95% confidence intervals is provided for each scoring system to evaluate and compare discriminative performance for in-hospital mortality.

In the overall cohort, ROC curve analysis showed that all five admission scoring systems demonstrated moderate discriminative ability for in-hospital mortality (Figure 8A). SOFA2 yielded the highest point-estimate AUC (0.837, 95% CI 0.743–0.932), followed closely by NEWS (0.835, 95% CI 0.758–0.912) and MEWS (0.820, 95% CI 0.737–0.902), whereas SOFA1 (0.800, 95% CI 0.691–0.909) and HSSS (0.794, 95% CI 0.682–0.907) showed broadly comparable performance. Overall, discrimination estimates were similar across scores, with substantial overlap in confidence intervals.

After subtype classification, discriminative performance was further evaluated within each subtype (Figures 8B,C). In Subtype 0, MEWS and NEWS showed relatively better discrimination, whereas SOFA2, SOFA1, and HSSS demonstrated limited separation (Figure 8B). However, mortality events were infrequent in this subtype, resulting in wide confidence intervals; these estimates should therefore be interpreted cautiously.

In Subtype 1, discrimination was more consistent across scoring systems (Figure 8C). SOFA2 yielded the highest point-estimate AUC, while NEWS, MEWS, SOFA1, and HSSS demonstrated moderate performance, indicating retained risk stratification capacity in this higher-risk context.

Decision curve analysis was subsequently performed in Subtype 1 (*n* = 136) (Supplementary Figure 6). Across a range of threshold probabilities, all scores yielded positive net benefit compared with default strategies. SOFA2 maintained relatively stable net benefit across most thresholds.

Given the limited number of deaths in Subtype 0, DCA results for this subgroup were not formally interpreted.

### 3.6 Secondary analyses of prolonged hospitalization among survivors

To complement the primary outcome analyses, exploratory secondary analyses were conducted among patients who survived to hospital discharge, after excluding those who died during hospitalization. These analyses aimed to evaluate the association between baseline disease severity at admission and subsequent hospitalization burden, as reflected by prolonged length of stay.

**Figure 9:**
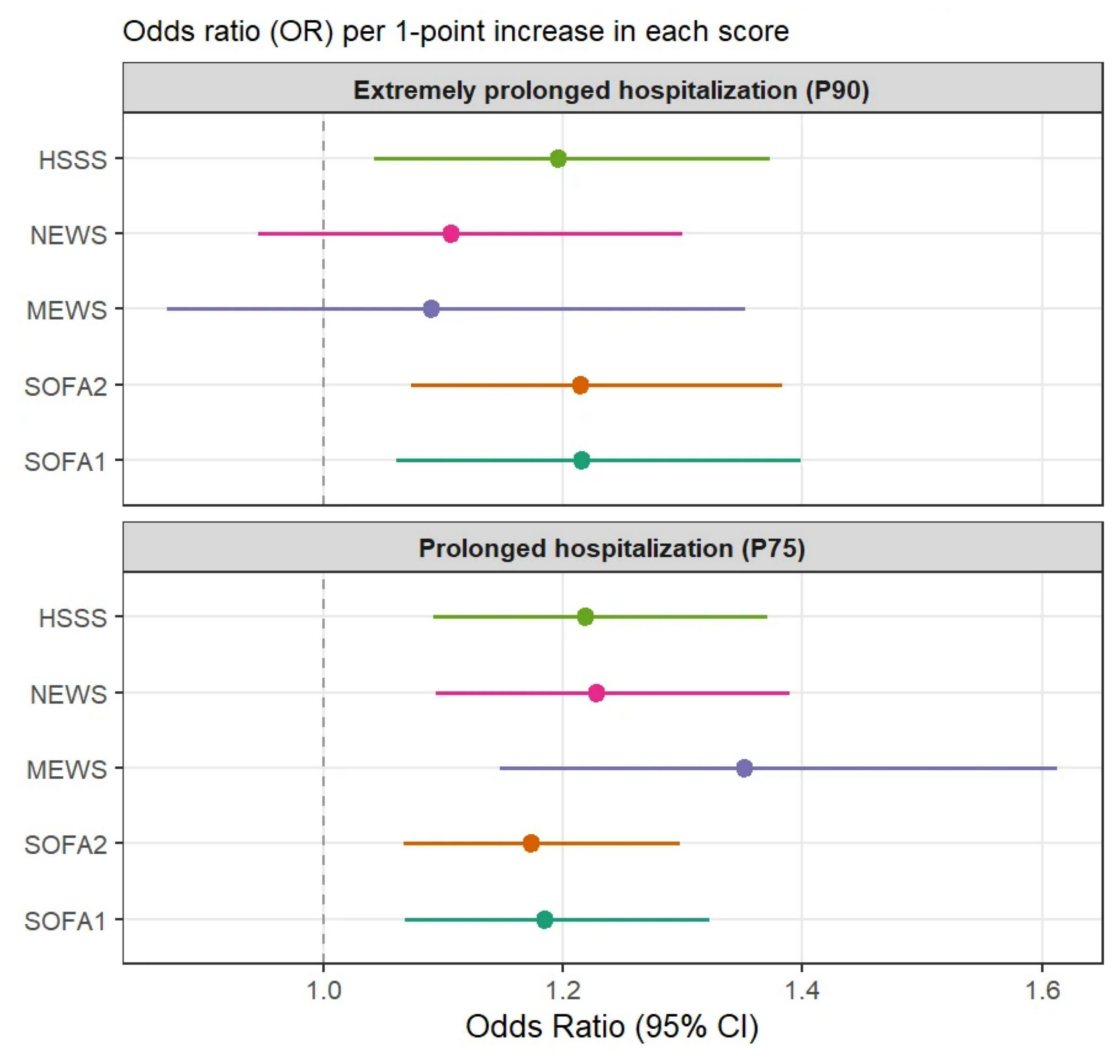
Forest plot of logistic regression analyses for prolonged hospitalization among survivors using different clinical scoring systems. This figure presents the results of univariable logistic regression analyses evaluating the associations between different clinical scoring systems (SOFA1, SOFA2, MEWS, NEWS, and HSSS) and prolonged hospitalization (P75) as well as extremely prolonged hospitalization (P90) among patients discharged alive. The x-axis represents the odds ratio (OR), and the y-axis lists the scoring systems. Point estimates indicate OR values, and horizontal lines denote the corresponding 95% confidence intervals. The dashed vertical line indicates the null value (OR = 1). The forest plot is divided into two panels, corresponding to prolonged hospitalization (P75) and extremely prolonged hospitalization (P90), respectively. OR values greater than 1 indicate an increasing risk of prolonged length of stay per 1-point increase in the score.

Results from univariable logistic regression analyses are shown in Figure 9 and Supplementary Table 3. Across all severity scoring systems, baseline scores demonstrated directionally consistent associations with both prolonged hospitalization (P75) and extremely prolonged hospitalization (P90), such that higher scores were generally associated with increased odds of prolonged length of stay.

ROC curve analyses for prolonged hospitalization outcomes are presented in Supplementary Figure 4. For both prolonged (P75) and extremely prolonged (P90) hospitalization, AUC estimates for the different scoring systems were overall in the moderate range, with relatively limited differences observed between scores. For the extremely prolonged hospitalization outcome (P90), SOFA2 showed a relatively higher point estimate of AUC; however, overall discriminative performance remained modest and should be interpreted with caution.

Overall, baseline severity scores at admission were able to reflect, to some extent, the risk trend for prolonged hospitalization among survivors, but their discriminative ability for length-of-stay–related outcomes was limited. These findings are presented as supplementary observations to the primary analyses of in-hospital mortality.

Subtype-specific discrimination analyses should be interpreted cautiously. Only 3 in-hospital deaths occurred in Subtype 0 and 21 in Subtype 1; therefore, these analyses were intended primarily as descriptive/exploratory assessments rather than definitive inferential comparisons (Supplementary Table 4).

## 4 Discussion

Heat stroke represents a systemic inflammatory syndrome rather than isolated hyperthermia, in which organ dysfunction scoring systems carry clear biological relevance. However, the applicability of existing severity scores for risk stratification in heat stroke—particularly in its severe forms characterized by extreme heat stress and synchronous multi-organ injury—remains uncertain in real-world, heterogeneous clinical settings.

Using a multicenter real-world cohort, this study systematically evaluated the risk characterization performance of SOFA2 and commonly used severity scores under a competing-risk framework, while further integrating data-driven phenotyping to account for patient heterogeneity. SOFA2 demonstrated a stable and continuous association with in-hospital mortality. At the overall cohort level, all five scoring systems showed broadly comparable discrimination, with SOFA2 yielding the highest point-estimate AUC but with substantial overlap in confidence intervals. In subtype-based analyses, discriminative performance appeared more consistent in the higher-risk clinical subtype identified through unsupervised clustering (Figures 5, 8; Supplementary Figure 6).

### 4.1 Main findings and pathophysiological interpretation

The primary contribution of this study is not to establish the categorical superiority of a single scoring system, but to demonstrate that score performance depends on clinical context and the extent to which organ dysfunction signals are manifest at admission. Severe heat stroke, triggered by extreme thermal stress, may rapidly progress through systemic inflammation, endothelial injury, and microcirculatory dysfunction, ultimately leading to multi-organ failure and increased short-term mortality risk (1–4). Coagulation abnormalities frequently evolve in parallel with organ injury and may further amplify adverse outcomes (5).

From a pathophysiological perspective, severe heat stroke shares substantial overlap with shock- and sepsis-related pathways, including disruption of intestinal barrier integrity, endotoxin translocation, release of damage-associated molecular patterns such as HMGB1, and activation of immunothrombotic mechanisms (5, 30–32). In this context, severity scores integrating multi-organ dysfunction may be particularly informative for short-term risk stratification.

Consistent with this framework, SOFA2 showed a stable positive association with in-hospital mortality under a competing-risk model, with restricted cubic spline analyses demonstrating a generally monotonic risk gradient (Figure 5). This finding aligns with the design rationale of SOFA2, which recalibrates original SOFA thresholds to achieve a more continuous relationship between score values and mortality risk (8). Compared with SOFA1, SOFA2 showed clearer stepwise separation of cumulative mortality risk across quartiles, particularly in intermediate risk ranges (Figure 2), suggesting potential advantages of threshold recalibration in noninfectious, high-stress critical illness.

### 4.2 Patient heterogeneity and differential score performance

Patients with heat stroke exhibit substantial heterogeneity in clinical presentation, disease stage at admission, and patterns of organ dysfunction. Unsupervised, data-driven phenotyping has increasingly been applied in critical care research to identify latent clinical subtypes with distinct physiological profiles and outcome risks without reliance on prespecified thresholds (21, 22). In the present study, two major clinical subtypes were identified based on early clinical and laboratory features, with marked differences in organ injury patterns and in-hospital mortality risk (Figures 6, 7). This stratified structure provides a clinically interpretable framework for understanding differential performance of severity scoring systems in heterogeneous patient populations.

Importantly, these subtypes were not generated from SOFA2 values, other composite severity scores, outcome variables, or treatment-/organ support–related indicators, all of which were excluded from the clustering feature matrix to reduce circularity. Nonetheless, because the retained early physiological and laboratory variables still capture manifestations of organ injury and systemic stress, we cannot fully exclude the possibility that the identified structure partly reflects a severity continuum. Accordingly, we interpret these groups primarily as data-driven early clinical subtypes or risk contexts, rather than as definitively established pathophysiological endotypes.

From a clinical perspective, the higher-risk subtype (Subtype 1) was characterized by predominant multi-organ dysfunction, including stronger circulatory stress signals, elevated hepatic injury markers, and abnormalities in coagulation- and platelet-related parameters. This constellation of features is consistent with prior evidence linking liver injury, coagulopathy, and circulatory dysfunction to adverse outcomes in heat stroke (35–37), and aligns with the core organ injury patterns emphasized in the most recent Society of Critical Care Medicine guidelines for the management of heat stroke (33, 34). In contrast, the lower-risk subtype (Subtype 0) displayed physiological and laboratory profiles closer to normal ranges, suggesting an earlier disease stage or less overt organ dysfunction at admission. Although these subtype differences may partly reflect a severity gradient, the clustering procedure excluded outcome variables, treatment variables, and severity score outputs, supporting the interpretation that the identified subtypes capture broader early clinical configurations rather than score-defined strata alone.

Given that the risk representation capacity of a severity score depends on the type of information it integrates, these subtype-specific differences offer a biologically plausible explanation for heterogeneous score performance. Vital sign–based early warning scores may be more sensitive to early physiological decompensation, whereas organ dysfunction–based scores rely on the explicit manifestation of laboratory abnormalities (15). In the present study, mortality events were sparse in Subtype 0, resulting in substantial uncertainty in AUC estimates, whereas in the higher-risk subtype, multiple scoring systems demonstrated moderate discriminative ability, with SOFA2 showing one of the highest point-estimate AUCs and relatively stable net benefit trends (Figure 8, Supplementary Figure 6).

Collectively, these findings suggest that differences in score performance among patients with heat stroke are more likely to reflect variation in underlying risk composition and disease stage rather than intrinsic superiority or inferiority of specific scoring systems. Accordingly, reliance on overall cohort-level performance metrics alone may obscure clinically meaningful, context-dependent information. This framework may also help explain previously reported inconsistencies in the prognostic performance of severity scores among patients with heat stroke or severe heat stroke across different studies (11).

### 4.3 Implications for clinical decision support

Clinical management of heat stroke is highly time sensitive, and the primary value of early risk stratification lies in informing decisions regarding ICU admission, monitoring intensity, and prioritization of healthcare resources. Importantly, the clinical utility of a severity score should not be equated solely with statistical discrimination, but interpreted in conjunction with potential clinical usefulness. In this context, decision curve analysis provides a pragmatic framework for comparing net benefit across a range of threshold probabilities (16–18, 29).

In the present study, SOFA2 demonstrated a stable, continuous positive association with in-hospital mortality (Figure 5). At the overall cohort level, discrimination was broadly comparable across scoring systems, although SOFA2 yielded the highest point-estimate AUC. In the higher-risk clinical subtype, SOFA2 also showed relatively stable net benefit trends (Figure 8, Supplementary Figure 6). These findings do not imply that SOFA2 is universally superior to other scoring systems, but rather suggest that when patients already exhibit overt organ dysfunction, integrated multi-organ scores may provide risk gradients more closely aligned with short-term adverse outcomes. This observation is consistent with prior reports indicating that SOFA-based scores tend to perform more stably in populations with higher baseline risk (7–15).

It should be emphasized that decision curve analyses in this study were based on univariable models and were intended to compare relative net benefit trends across scoring systems within the same patient set, rather than to define intervention thresholds or support causal inference (29). Accordingly, SOFA2 should be interpreted as a tool for risk representation and stratification rather than a standalone decision-making instrument.

In clinical practice, early SOFA2 assessment within the first 24 h of admission may support prioritization of intensive monitoring, organ function surveillance, and allocation of critical care resources. At the research level, SOFA2-based stratification may facilitate risk enrichment in prospective studies or interventional trials, potentially improving efficiency in detecting outcome differences. This concept aligns with enrichment strategies recommended by regulatory agencies, including the U.S. Food and Drug Administration, for clinical trial design (38, 39). Nevertheless, clinical application should always be integrated with etiological assessment, dynamic reassessment, and prospective validation.

### 4.4 Relationship to previous studies

Most previous studies evaluating prognosis in patients with heat stroke or severe heat stroke have focused on single scoring systems or single-center cohorts, and have predominantly relied on Kaplan–Meier or Cox proportional hazards models (9–11). In the context of in-hospital mortality, discharge alive represents a competing event rather than a noninformative censoring process, as lower-risk patients are more likely to be discharged earlier and removed from the risk set. Failure to account for this competing process may bias absolute risk estimates and distort comparisons between severity scoring systems (40, 41).

In the present study, multiple severity scores were compared within a competing-risk framework, and restricted cubic splines were used to characterize continuous risk gradients across the score spectrum. Under this approach, SOFA2 demonstrated a stable monotonic association with in-hospital mortality, with no evidence of substantial nonlinearity, consistent with its design rationale of improved risk continuity through threshold recalibration (8) (Figures 2, 5). These findings were robust in the ICU subgroup.

In addition, by integrating unsupervised data-driven phenotyping, we demonstrated substantial heterogeneity among patients with heat stroke and observed that score performance varied across clinical subtypes. This observation parallels prior findings in ARDS and sepsis populations, where subtype-dependent differences in prognostic performance have been reported (21, 22), and offers a potential structural explanation for inconsistencies observed across previous heat stroke studies.

### 4.5 Additional considerations and limitations

Beyond short-term mortality, we further explored associations between admission severity scores and prolonged length of hospital stay among survivors as supplementary analyses. Although higher baseline scores were directionally associated with prolonged and extremely prolonged hospitalization, discriminative performance for length-of-stay–related outcomes remained modest (Figure 9, Supplementary Figure 4). This pattern is consistent with prior evidence indicating that hospital length of stay is shaped not only by disease severity, but also by healthcare processes, bed availability, and social factors (25–28). Consequently, even severity scores that demonstrate stable performance for mortality risk stratification may have limited utility in discriminating hospitalization burden. These findings underscore the importance of interpreting length-of-stay analyses as complementary rather than primary indicators of clinical utility.

Several limitations should be considered when interpreting the overall findings of this study. First, the retrospective observational design is inherently subject to selection and information bias, and observed associations should not be interpreted causally.

Second, the number of in-hospital deaths was limited (24/292 overall), particularly after stratification by clinical subtype (21 deaths in Subtype 1 and 3 deaths in Subtype 0). Although the overall analytic framework was pre-specified, the low event count constrained statistical power relative to the analytic complexity, especially for spline-based models and subtype-stratified discrimination analyses. In the overall cohort, the minimally adjusted regression models remained relatively parsimonious (approximately 12 events per variable for linear score + age models and approximately 8 events per effective degree of freedom for restricted cubic spline models), but precision was substantially reduced in subtype-specific analyses. Accordingly, subtype-specific ROC, DCA, and related comparative findings should be interpreted as exploratory and hypothesis-generating rather than definitive evidence of differential score performance.

Third, this cohort was derived from two tertiary hospitals in China, and external generalizability should be interpreted with caution. Importantly, in accordance with the ethics and confidentiality framework of this study, the cohort did not include military-related personnel. In addition, only 27 patients were classified as exertional heat stroke, while the cohort also included pediatric and older patients (10.3% aged < 18 years and 13.3% aged >50 years), indicating that the study population was not restricted to a narrowly defined young exertional subgroup. Nevertheless, because the study was conducted in two centers within a single national setting, external validation remains necessary in independent cohorts with different environmental exposures, age structures, and clinical practice patterns, including populations with a higher proportion of classic heat stroke.

Fourth, although multivariable imputation was applied for phenotyping and complete-case analysis was used for decision curve evaluation, residual bias related to missing data cannot be fully excluded. In addition, SOFA2 permits limited substitute scoring under the official consensus algorithm, whereas the other four scores were calculated strictly according to their original rules. In our cohort, however, such SOFA2 substitution was required in only one patient, and all five scores were available in the same 292 patients; therefore, the likelihood that differential score availability materially influenced the comparative results is likely limited, although this possibility cannot be completely excluded.

In addition, although the clustering procedure excluded severity score outputs, outcomes, and treatment-related variables, the retained early physiological and laboratory features may still partly encode overall illness severity. Therefore, the identified subtypes should not be overinterpreted as definitively distinct biological phenotypes without external validation and replication.

Finally, this study focused primarily on discrimination and potential decision support value; systematic evaluation of model calibration and threshold selection was beyond its scope and should be addressed in future external validation studies (42).

## 5 Conclusion

In this multicenter retrospective cohort of patients with heat stroke, the SOFA 2.0 score showed a stable positive association with in-hospital mortality and demonstrated clear, continuous risk gradients at early hospital admission. By integrating data-driven phenotyping, we observed that SOFA2 showed numerically more consistent discriminative performance and potential net benefit trends in a higher-risk clinical subtype; however, these subtype-level findings should be regarded as exploratory, and the identified subtype structure may partly overlap with illness severity. These findings suggest that SOFA2 may serve as a useful tool for early risk stratification in patients with heat stroke, particularly when organ dysfunction is evident. External validation in independent cohorts is required before broader clinical implementation.

## Data Availability

The raw data supporting the conclusions of this article will be made available by the authors, without undue reservation.

## References

[1] Bouchama A Knochel JP. Heat stroke. N Engl J Med. (2002) 346:1978–88. doi: 10.1056/NEJMra011089 12075060

[2] Epstein Y Roberts WO. The pathopysiology of heat stroke: an integrative view of the final common pathway. Scand J Med Sci Sports. (2011) 21:742–8. doi: 10.1111/j.1600-0838.2011.01333.x 21635561

[3] Leon LR Bouchama A. Heat stroke. Compr Physiol. (2015) 5:611–47. doi: 10.1002/j.2040-4603.2015.tb00612.x 25880507

[4] Bouchama A Abuyassin B Lehe C Laitano O Jay O O'Connor FG . Classic and exertional heatstroke. Nat Rev Dis Primers. (2022) 8:8. doi: 10.1038/s41572-021-00334-6 35115565

[5] Iba T Connors JM Levi M Levy JH. Heatstroke-induced coagulopathy: biomarkers, mechanistic insights, and patient management. EClinicalMedicine. (2022) 44:101276. doi: 10.1016/j.eclinm.2022.101276 35128366 PMC8792067

[6] Liu SY Song JC Mao HD Zhao JB Song Q Expert Expert Group of Heat Stroke Prevention and Treatment of the People's Liberation Army and and People's Liberation Army Professional Committee of Critical Care Medicine. Expert consensus on the diagnosis and treatment of heat stroke in China. Mil Med Res. (2020) 7:1. doi: 10.1186/s40779-019-0229-2 31928528 PMC6956553

[7] Kashyap R Sherani KM Dutt T Gnanapandithan K Sagar M Vallabhajosyula S . Current utility of Sequential Organ Failure Assessment score: a literature review and future directions. Open Respir Med J. (2021) 15:1–6. doi: 10.2174/1874306402115010001 34249175 PMC8227444

[8] Moreno R Rhodes A Ranzani O Salluh JIF Berger-Estilita J Coopersmith CM . Rationale and methodological approach underlying the development of the Sequential Organ Failure Assessment (SOFA)-2 score: a consensus statement. JAMA Netw Open. (2025) 8:e2545040. doi: 10.1001/jamanetworkopen.2025.45040 41159829

[9] Yokoyama K Kaneko T Ito A Ieki Y Kawamoto E Suzuki K . Sequential organ failure assessment score as a predictor of the outcomes of patients hospitalized for classical or exertional heatstroke. Sci Rep. (2022) 12:16373. doi: 10.1038/s41598-022-20878-1 36180581 PMC9525654

[10] Wu M Wang C Liu Z Liu Z. Sequential Organ Failure Assessment score for prediction of mortality of patients with rhabdomyolysis following exertional heatstroke: a longitudinal cohort study in Southern China. Front Med. (2021) 8:724319. doi: 10.3389/fmed.2021.724319 34708052 PMC8542709

[11] Subbe CP Kruger M Rutherford P Gemmel L. Validation of a modified early warning score in medical admissions. QJM. (2001) 94:521–6. doi: 10.1093/qjmed/94.10.521 11588210

[12] Royal College of Physicians. National Early Warning Score 2 (NEWS2): Standardising the Assessment of Acute-Illness Severity in the NHS. London: RCP (2017).

[13] Smith ME Chiovaro JC O'Neil M Kansagara D Quiñones AR Freeman M . Early warning system scores for clinical deterioration in hospitalized patients: a systematic review. Ann Am Thorac Soc. (2014) 11:1454–65. doi: 10.1513/AnnalsATS.201403-102OC 25296111

[14] Cox DR. Regression models and life-tables. J R Stat Soc Ser A (1972) 34:187–202. doi: 10.1111/j.2517-6161.1972.tb00899.x

[15] Hanley JA McNeil BJ. The meaning and use of the area under a receiver operating characteristic (ROC) curve. Radiology. (1982) 143:29–36. doi: 10.1148/radiology.143.1.7063747 7063747

[16] Vickers AJ Elkin EB. Decision curve analysis: a novel method for evaluating prediction models. Med Decis Making. (2006) 26:565–74. doi: 10.1177/0272989X06295361 17099194 PMC2577036

[17] Vickers AJ Van Calster B Steyerberg EW. Net benefit approaches to the evaluation of prediction models, molecular markers, and diagnostic tests. BMJ. (2016) 352:i6. doi: 10.1136/bmj.i6 26810254 PMC4724785

[18] McLachlan GJ Peel D. Finite Mixture Models. New York: Wiley (2000). ISBN: 978-0-471-00626-8.

[19] Jolliffe IT. Principal Component Analysis. 2nd ed. New York: Springer (2002). ISBN: 978-0-387-95442-4.

[20] Schwarz G. Estimating the dimension of a model. Ann Stat. (1978) 6:461–4. doi: 10.1214/aos/1176344136

[21] Calfee CS Delucchi K Parsons PE Thompson BT Ware LB Matthay MA . Subphenotypes in acute respiratory distress syndrome: latent class analysis of data from two randomised controlled trials. Lancet Respir Med. (2014) 2:611–20. doi: 10.1016/S2213-2600(14)70097-9 24853585 PMC4154544

[22] Seymour CW Kennedy JN Wang S Chang CH Elliott CF Xu Z . Derivation, validation, and potential treatment implications of novel clinical phenotypes for sepsis. JAMA. (2019) 321:2003–17. doi: 10.1001/jama.2019.5791 31104070 PMC6537818

[23] von Elm E Altman DG Egger M Pocock SJ Gøtzsche PC Vandenbroucke JP . The strengthening the reporting of observational studies in epidemiology (STROBE) statement: guidelines for reporting observational studies. Lancet. (2007) 370:1453–7. doi: 10.1016/S0140-6736(07)61602-X 18064739

[24] Collins GS Reitsma JB Altman DG Moons KG. Transparent reporting of a multivariable prediction model for individual prognosis or diagnosis (TRIPOD): the TRIPOD statement. Ann Intern Med. (2015) 162:55–63. doi: 10.7326/M14-0697 25560714

[25] Stone K Zwiggelaar R Jones P Mac Parthaláin N. A systematic review of the prediction of hospital length of stay: towards a unified framework. PLOS Digit Health. (2022) 1:e0000017. doi: 10.1371/journal.pdig.0000017 36812502 PMC9931263

[26] Tipton K Leas BF Mull NK Siddique SM Greysen SR Lane-Fall MB . Interventions to Decrease Hospital Length of Stay. Rockville, MD, USA: Agency for Healthcare Research and Quality (2021). 34644039

[27] Siddique SM Tipton K Leas B Greysen SR Mull NK Lane-Fall M . Interventions to reduce hospital length of stay in high-risk populations: a systematic review. JAMA Netw Open. (2021) 4:e2125846. doi: 10.1001/jamanetworkopen.2021.25846 34542615 PMC8453321

[28] Steyerberg EW Vickers AJ Cook NR Gerds T Gonen M Obuchowski N . Assessing the performance of prediction models: a framework for traditional and novel measures. Epidemiology. (2010) 21:128–38. doi: 10.1097/EDE.0b013e3181c30fb2 20010215 PMC3575184

[29] Sadatsafavi M Adibi A Puhan M Gershon A Aaron SD Sin DD. Moving beyond AUC: decision curve analysis for quantifying net benefit of risk prediction models. Eur Respir J. (2021) 58:2101186. doi: 10.1183/13993003.01186-2021 34503984

[30] Sun M Li Q Zou Z Liu J Gu Z Li L. The mechanisms behind heatstroke-induced intestinal damage. Cell Death Discov. (2024) 10:455. doi: 10.1038/s41420-024-02210-0 39468029 PMC11519599

[31] Iba T Helms J Levi M Levy JH. The role of platelets in heat-related illness and heat-induced coagulopathy. Thromb Res. (2023) 231:152–8. doi: 10.1016/j.thromres.2022.08.009 35989192

[32] Zhang Y Deng X Zhang J Zhang L Akram Z Zhang B . A potential driver of disseminated intravascular coagulation in heat stroke mice: neutrophil extracellular traps. Int J Environ Res Public Health. (2022) 19:12448. doi: 10.3390/ijerph191912448 36231751 PMC9566744

[33] Barletta JF Palmieri TL Toomey SA AlShamsi F Stearns RL Patanwala AE . Society of Critical Care Medicine guidelines for the treatment of heat stroke. Crit Care Med. (2025) 53:e490–500. doi: 10.1097/CCM.0000000000006551 39982186

[34] Barletta JF Palmieri TL Toomey SA AlShamsi F Stearns RL Patanwala AE . Executive summary: Society of Critical Care Medicine guidelines for the treatment of heat stroke. Crit Care Med. (2025) 53:e483–9. doi: 10.1097/CCM.0000000000006550 39982185

[35] Feng L Yin JY Liu YH Zhang P Zhao YL Song Q . N-terminal pro-brain natriuretic peptide: a significant biomarker of disease development and adverse prognosis in patients with exertional heat stroke. Mil Med Res. (2024) 11:26. doi: 10.1186/s40779-024-00531-w 38654334 PMC11036771

[36] Wang F Wu K Shi X Xie L Lin G Li J . Liver enzyme trajectory and risk factors for acute liver failure in patients with exertional heatstroke: a retrospective analysis. Intern Emerg Med. (2026). doi: 10.1007/s11739-025-04245-2 41501508

[37] Moreno R Rhodes A Piquilloud L Hernandez G Takala J Gershengorn HB . The Sequential Organ Failure Assessment (SOFA) score: has the time come for an update? Crit Care. (2023) 27:15. doi: 10.1186/s13054-022-04290-9 36639780 PMC9837980

[38] Kent DM Hayward RA. Limitations of applying summary results of clinical trials to individual patients: the need for risk stratification. JAMA. (2007) 298:1209–12. doi: 10.1001/jama.298.10.1209 17848656

[39] U.S. Food and Drug Administration. Enrichment Strategies for Clinical Trials to Support Determination of Effectiveness of Human Drugs and Biological Products: Guidance for Industry (2019). Available online at: https://www.fda.gov/regulatory-information/search-fda-guidance-documents/enrichment-strategies-clinical-trials-support-approval-human-drugs-and-biological-products (Accessed April 10, 2026).

[40] Fine JP Gray RJ. A proportional hazards model for the subdistribution of a competing risk. J Am Stat Assoc. (1999) 94:496–509. doi: 10.1080/01621459.1999.10474144

[41] Lau B Cole SR Gange SJ. Competing risk regression models for epidemiologic data. Am J Epidemiol. (2009) 170:244–56. doi: 10.1093/aje/kwp107 19494242 PMC2732996

[42] Austin PC Fine JP. Practical recommendations for reporting Fine-Gray model analyses for competing risk data. Stat Med. (2017) 36:4391–400. doi: 10.1002/sim.7501 28913837 PMC5698744

