# Supplementary material for: Predictive performance of the SOFA 2.0 score for in-hospital mortality in patients with heat stroke: a multicenter, data-driven subphenotype study
Source: Front Med (Lausanne). 2026 Apr 13;13:1818390. doi: 10.3389/fmed.2026.1818390 (PMC13111213; doi:10.3389/fmed.2026.1818390)
Supplement: Supplementary file 1 [file Data_Sheet_1.pdf]

Supplementary Materials for

Predictive Performance of the SOFA 2.0 Score for In-Hospital Mortality in Patients With Heat Stroke: A Multicenter, Data-Driven Subphenotype Study

Haoming Luo, Yirui Zhu, Tongtong Wang, Weihua Li, Haiyang Guo, Chen Ting, Yuanyuan Ou,Mengshan Guan,Yifeng Zhang,Guoxuan Lin.
Correspondence to: Zhiguo Pan(pzgsubject@126.com); Anwei Liu (anweiliu2014@163.com).

The First Clinical Medical School of Guangdong Pharmaceutical University

This file includes:

Supplementary Methods
Tables S1 to S4
Figures S1 to S6

Supplementary Methods

1 Detailed Supplementary Description of Data Screening, Variable Processing, and Analytical Workflow

To enhance the transparency and reproducibility of the study, we provide additional details on the procedures for data screening, variable processing, score calculation, and phenotyping analyses, based on the Materials and Methods section of the main text. The overall analytical workflow is summarized below:

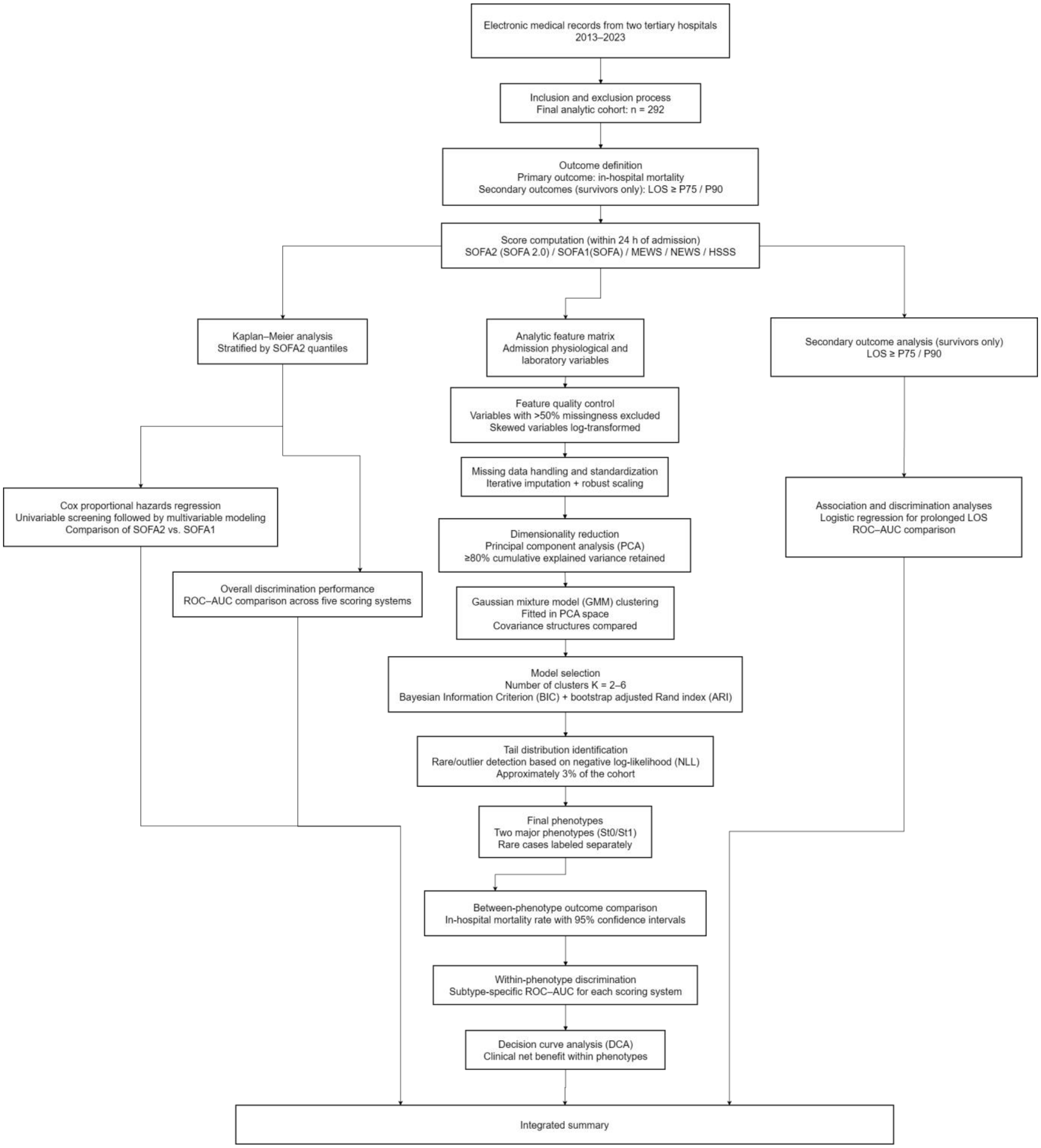

1.1 Study population screening and data sources

This study was based on electronic medical record (EMR) data of patients with heat stroke admitted between 2013 and 2023 from two tertiary hospitals in China. The initial search included all patients admitted through the emergency department with a diagnosis of heat stroke or heat-related illness, without age restriction. The initial inclusion criteria were as follows: (1) first emergency admission with a diagnosis related to heat stroke; and (2) complete laboratory and examination results available within the first 24 hours after admission. A total of 448 patients met these criteria and were included in the initial screening cohort.

Based on this cohort, we extracted the first available baseline demographic characteristics, vital signs, laboratory measurements, and variables necessary for severity score calculation at admission. To ensure data integrity and analytical robustness, patients with missing primary outcomes or with ≥10% missingness in essential variables during the first 24 hours after admission were excluded. A total of 66 patients met the exclusion criteria. The final analysis incorporated 51 predefined clinically relevant variables spanning demographic information, physiological measurements, laboratory parameters, organ dysfunction indicators, and treatment interventions.

1.2 Handling of missing key variables and outliers

In the remaining cohort, we further evaluated the completeness and numerical plausibility of variables required for score calculation and core laboratory indicators. Patients with missing key variables required for severity score computation, or with extreme values exceeding twice the physiological range without clinically explainable evidence during manual data verification after extraction, were excluded if such values could not be reasonably corrected or substituted. A total of 88 patients were excluded based on these criteria. Ultimately, 292 patients were included in the final analytical cohort.

The above screening and exclusion procedures were primarily driven by data availability and analytical feasibility and were not based on outcome information; therefore, they did not constitute prognosis-driven selective exclusion.

1.3 Score calculation and variable standardization

In the final analytical cohort, SOFA 2.0 (SOFA2) was calculated according to the prespecified definition. The SOFA2 score incorporated baseline organ dysfunction components and was further assessed using the first available supportive information after admission (e.g., vasopressor use and advanced respiratory support). In addition, the traditional SOFA score (SOFA1) was calculated according to its original definition for comparative analyses.

To facilitate subsequent statistical analyses and model comparisons, necessary unit harmonization and standardization were performed for selected continuous variables before inclusion in the models.

1.4 Unsupervised phenotyping analysis

In the unsupervised phenotyping analysis, only variables reflecting early physiological status and laboratory characteristics at admission were included. Outcome variables, severity score results, and treatment- or organ support–related indicators (e.g., mechanical ventilation, vasopressor use, and renal replacement therapy [RRT]) were explicitly excluded to reduce the risk of information leakage and circular reasoning.

The phenotyping feature matrix was constructed using the first available physiological and laboratory measurements obtained within 24 hours of admission. During preprocessing, candidate variables with a missing rate >50% were removed. Highly skewed laboratory variables were log-transformed, whereas other continuous variables were uniformly scaled using robust standardization (RobustScaler) to reduce the influence of extreme values on clustering structure. Missing values in retained variables were imputed using multivariable iterative imputation (Iterative Imputer). This imputation procedure was applied solely for construction of the phenotyping feature matrix and was not used for severity score calculation or for regression models evaluating the primary outcomes.

Principal component analysis (PCA) was then applied to the standardized feature space for dimensionality reduction. A total of 11 principal components were retained, explaining 81.7% of the cumulative variance. Subsequently, Gaussian mixture models (GMMs) were fitted in the PCA space, and different numbers of clusters (K=2–6) and covariance structures were systematically compared.

Model selection was primarily based on the Bayesian information criterion (BIC), with an additional stability penalty incorporating the bootstrap-derived adjusted Rand index (ARI), aiming to balance model fit and reproducibility. Based on the overall evaluation, a GMM with diagonal covariance structure (diag) and K=2 was selected as the primary phenotyping solution. To further assess the robustness of the phenotyping structure, clustering consistency was validated using bootstrap resampling and 80% subsampling without replacement, and a consensus clustering matrix was generated to visualize cluster stability. Relevant results are presented in the main text (Fig 6).

1.5 Outcome analysis and evaluation of predictive performance

After phenotyping, SOFA2, traditional SOFA (SOFA1), MEWS, NEWS, and HSSS were calculated, and their prognostic performance for in-hospital mortality was evaluated in the overall cohort and within each clinical subtype. Discriminative ability across scoring systems was compared using receiver operating characteristic (ROC) curves and the area under the curve (AUC). Decision curve analysis (DCA) was further performed to assess the net clinical benefit of each scoring system across a range of threshold probabilities.

1.6 Code availability and implementation details

All statistical analyses and model development were performed in R and Python under fixed random seed settings to ensure reproducibility.

Conventional statistical analyses, including descriptive statistics, survival analyses, regression modeling, and decision curve analysis, were conducted in R (version 4.5.2), primarily using commonly applied packages such as survival, survminer, tableone, gtsummary, and rmda.

Unsupervised phenotyping analyses were conducted in Python (version 3.10.19). Data preprocessing and numerical computation were performed using pandas and numpy. Missing values were imputed using multivariable iterative imputation (IterativeImputer); notably, imputation was applied only to variables used for unsupervised phenotyping and was not performed for outcome variables or primary predictors in the main regression models. Robust standardization (RobustScaler) was applied to reduce the influence of extreme values. Dimensionality reduction was performed using principal component analysis (PCA), followed by fitting Gaussian mixture models (GMMs) in the PCA space, with systematic comparisons across different covariance structures (diag, tied, full) and numbers of clusters. Example code is provided below：

```
# Core unsupervised phenotyping workflow
X_cols, X_scaled = build_feature_matrix(df_raw, miss_thresh=0.50)

pca = PCA(n_components=0.80, random_state=42)
X_pca = pca.fit_transform(X_scaled)

metrics, models = fit_gmm_grid(X_pca, k_min=2, k_max=6, covariance_types=("diag","tied","full"))
_, _, best_model = select_best_model_no_tiny_stability_first(metrics, models, X_pca)

labels = best_model.predict(X_pca)
subtype_labels, _ = adaptive_merge_labels(labels, X_pca)
```

The final clustering solution was selected by jointly considering model fit and stability. Bayesian information criterion (BIC) served as the primary metric, and bootstrap-derived clustering stability indices (adjusted Rand index, ARI) were incorporated as a stability penalty term to balance goodness-of-fit and reproducibility. To mitigate potential distortion caused by extreme individuals, a two-stage outlier identification strategy based on individual negative log-likelihood (NLL) values was further applied. Approximately 3% of the cohort was flagged as rare/outlier samples, which were excluded from the construction of the primary clinical subtypes.

7. Statistical supplementary information

To further contextualize the regression-based analyses, univariable Cox proportional hazards models were additionally performed for all candidate baseline clinical variables. The full results of these analyses are summarized in Table S1, which presents unadjusted hazard ratios

(HRs) and corresponding 95% confidence intervals for each variable with respect to all-cause in-hospital mortality.

Table S1 serves as a comprehensive descriptive overview of the individual associations between admission characteristics, laboratory parameters, organ support indicators, and clinical scoring systems with mortality risk. These univariable results were not intended for causal inference, but rather to provide preliminary risk characterization and to inform subsequent model specification, variable selection, and interpretation of the multivariable Fine–Gray and Cox regression analyses reported in the main text.

To perform sensitivity analyses corresponding to the restricted cubic spline (RCS) curves derived from the Fine–Gray subdistribution hazard models, we additionally generated plots using conventional Cox proportional hazards regression (Supplementary Fig. S5). To evaluate the applicability of the Cox proportional hazards model, proportional hazards (PH) assumption testing was conducted for both the main linear models and the RCS-extended models.

PH assumption testing was performed using Schoenfeld residual analyses. The cox.zph() function (transform = "km") was applied to assess whether covariate effects exhibited systematic time-dependent variation. In Schoenfeld residual plots, time is displayed on the x-axis and the estimated time-varying regression coefficient $\beta(t)$ is shown on the y-axis. Each point represents the Schoenfeld residual at an event time, the solid line indicates the smoothed fitted curve, and the dashed lines represent the approximate 95% confidence interval. A relatively stable fitted curve fluctuating around 0 without an apparent monotonic trend suggests no evidence of violation of the PH assumption.

In the overall cohort, neither the linear SOFA model (SOFA + age) nor the RCS-extended model (SOFA + spline terms + age) demonstrated clear time-dependent effects. The fitted Schoenfeld residual curves remained generally stable, and the global PH test P values were high (all $P > 0.95$), indicating no significant violation of the PH assumption. The same procedures were repeated in the ICU subgroup. Similarly, in ICU-admitted patients, both the linear and RCS models for SOFA1 and SOFA2 showed no apparent time-dependent patterns; the Schoenfeld residual curves remained stable, and the proportional hazards (PH) assumption was not rejected (global P values all > 0.90). Detailed results are shown below:

① Full Cohort Cox Regression Model Schoenfeld

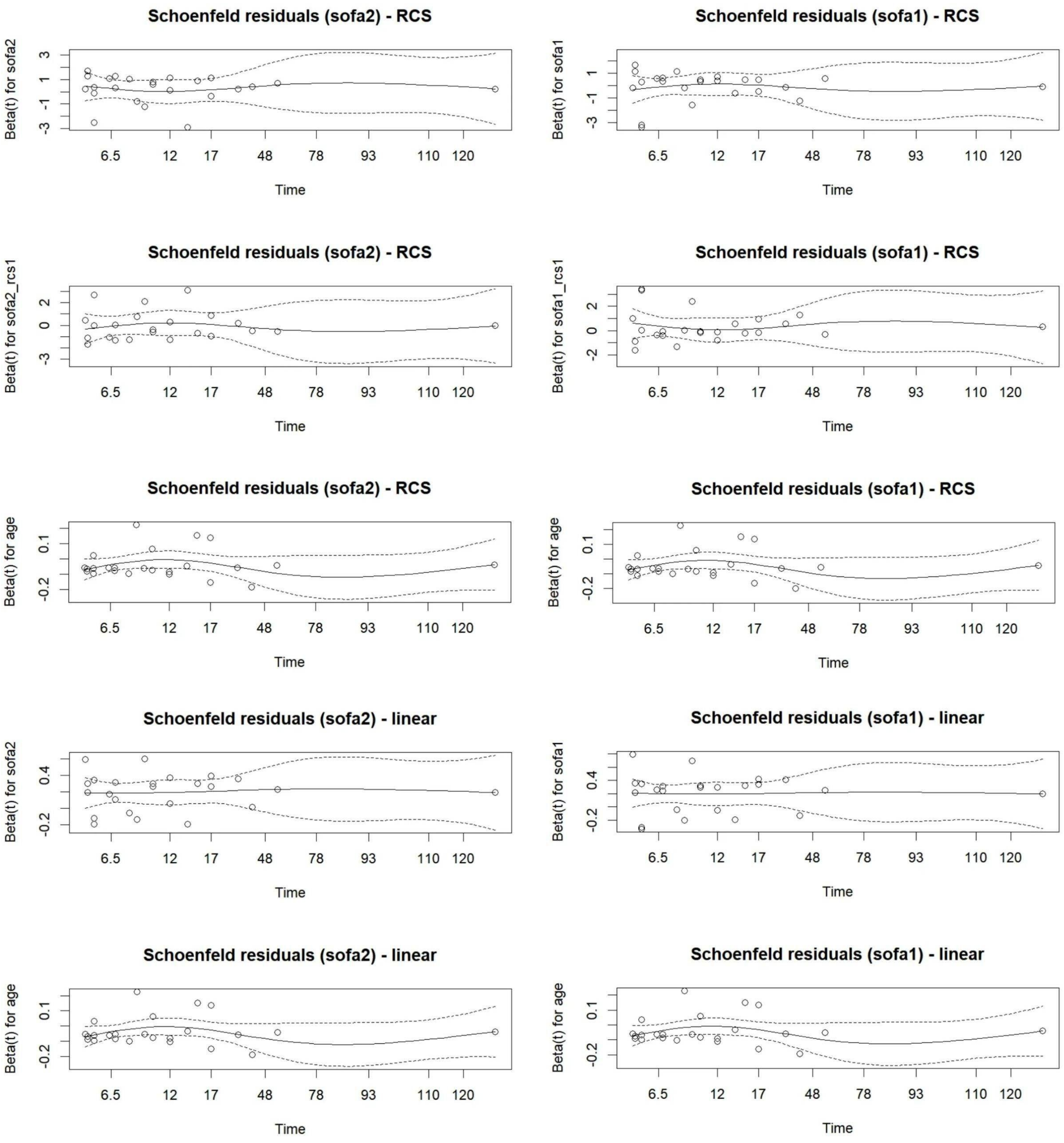

② Cox regression model for ICU subgroup with

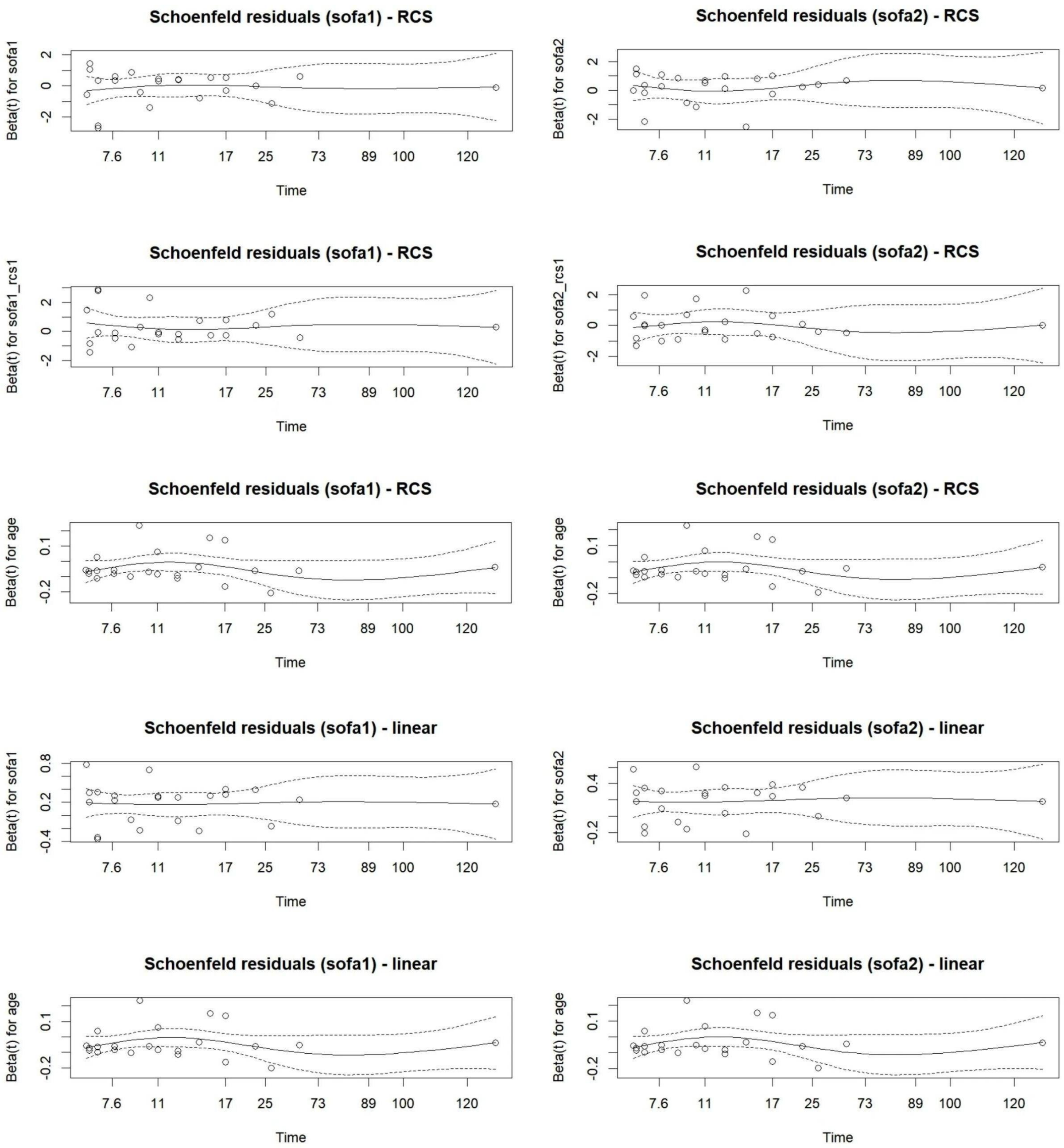

These findings suggest that the conventional Cox proportional hazards models satisfied the PH assumption in both the overall cohort and the ICU subgroup, supporting the statistical validity of the regression-based associations between SOFA1/SOFA2 scores and in-hospital mortality risk.

2 Supplementary Tables (S1 to S4)

Table.S2 Baseline characteristics independent of SOFA2 components

Characteristic	Overall	Low SOFA2	High SOFA2	|SMD|[2]	P value[3]
N	N = 293[1]	N = 162[1]	N = 131[1]		
Age, years	23.00 (20.00, 36.00)	22.00 (20.00, 29.00)	25.00 (20.00, 43.00)	0.27	0.018
Temperature, °C	37.00 (36.50, 37.80)	36.90 (36.50, 37.50)	37.20 (36.70, 38.20)	0.32	0.003
Heart rate, bpm	78.00 (67.00, 95.00)	75.00 (65.00, 87.00)	87.00 (72.00, 102.00)	0.56	<0.001
Respiratory rate, breaths/min	20.00 (18.00, 20.00)	20.00 (18.00, 20.00)	20.00 (19.00, 21.00)	0.25	0.082
Blood urea nitrogen, mmol/L	5.30 (3.70, 7.30)	4.70 (3.05, 6.10)	6.40 (4.70, 9.50)	0.69	<0.001
Sodium, mmol/L	142.00 (139.00, 144.00)	141.00 (139.00, 143.00)	142.00 (140.00, 145.00)	0.33	0.006
Potassium, mmol/L	3.70 (3.40, 4.00)	3.75 (3.50, 4.05)	3.70 (3.40, 4.00)	0.05	0.629
Calcium, mmol/L	2.13 (1.97, 2.27)	2.16 (2.03, 2.34)	2.05 (1.83, 2.21)	0.54	<0.001
Glucose, mmol/L	5.57 (4.82, 6.81)	5.40 (4.74, 6.50)	6.00 (4.90, 7.40)	0.24	0.041
Alanine aminotransferase, U/L	49.00 (21.00, 246.00)	30.00 (16.00, 65.00)	148.00 (33.00, 819.00)	0.61	<0.001
Aspartate aminotransferase, U/L	75.00 (30.00, 308.00)	38.50 (21.00, 96.00)	252.00 (75.00, 895.00)	0.49	<0.001
Creatine kinase, U/L	935.00 (275.00, 2,908.00)	508.00 (179.00, 1,621.00)	1,377.00 (696.00, 4,571.50)	0.09	<0.001
Lactate dehydrogenase, U/L	377.00 (243.00, 721.00)	286.50 (200.00, 386.50)	602.00 (372.00, 1,363.00)	0.75	<0.001
White blood cell count, ×10^9/L	10.29 (7.58, 13.65)	9.83 (7.23, 12.99)	11.46 (8.47, 14.53)	0.32	0.008
Procalcitonin, ng/mL	1.98 (0.81, 4.73)	1.28 (0.51, 3.37)	2.89 (1.22, 6.26)	0.09	<0.001
C-reactive protein, mg/L	4.90 (2.20, 14.50)	3.34 (1.02, 13.29)	6.80 (3.21, 18.80)	0.06	0.003
Prothrombin time, s	15.90 (14.10, 19.60)	15.00 (13.70, 16.40)	18.00 (15.40, 26.60)	0.64	<0.001
Activated partial thromboplastin time, s	38.95 (33.60, 45.00)	37.20 (32.70, 42.00)	40.50 (35.90, 58.10)	0.66	<0.001
D-dimer, mg/L	1.63 (0.48, 6.04)	0.65 (0.34, 2.30)	4.29 (1.44, 13.35)	0.13	<0.001

[1]Continuous variables are presented as median (IQR). SOFA2 groups were defined using the median cut-off (5). This table excludes SOFA2 component variables and all treatment/resource-use variables to avoid circularity. Between-group imbalance is assessed using absolute standardized mean differences (|SMD|). Wilcoxon rank-sum test was used for between-group comparisons.

[2]Standardized Mean Difference

[3]Wilcoxon rank sum test

Baseline admission characteristics are compared between the overall cohort and groups stratified by SOFA2 (low SOFA2 vs high SOFA2). SOFA2 grouping was defined using the median score as the cutoff. To avoid circular reasoning, variables constituting the SOFA2 score and treatment- or resource utilization–related indicators were not included in the table.

Continuous variables are presented as median (interquartile range [IQR]). Between-group differences were assessed using the Wilcoxon rank-sum test. The magnitude of baseline imbalance between groups was evaluated using the absolute standardized mean difference (SMD).

After excluding SOFA2 component variables and treatment-related indicators, patients in the high SOFA2 group still exhibited more pronounced abnormalities in multiple admission metabolic, hepatic and renal function, coagulation, and inflammation-related parameters. These findings suggest that SOFA2-based stratification reflects systematic differences in overall disease severity at early admission, rather than being driven solely by variables directly included in the score.

Table.S1 Univariable Cox regression analysis of all candidate covariates

Variable	HR (95% CI)	P value
hsss	1.222 (1.112–1.342)	<0.001
rrt_0_1	9.193 (3.115–27.133)	<0.001
Heart rate, bpm	1.036 (1.018–1.055)	<0.001
vasopressors_and_inotropes_0_1	6.552 (2.341–18.336)	<0.001
agi_grading_of_acute_gastrointestinal_injury_0_1	6.685 (2.258–19.793)	<0.001
Age, years	0.955 (0.923–0.987)	0.006
cr_umol_l	1.003 (1.001–1.005)	0.009
Creatinine, per 10 μmol/L	1.030 (1.008–1.053)	0.009
Mean arterial pressure, mmHg	0.968 (0.943–0.993)	0.012
plt_x109_l	0.995 (0.990–0.999)	0.024
Platelet count, per 10 ×10^9/L	0.948 (0.905–0.993)	0.024
spo2	0.964 (0.932–0.998)	0.04
SpO_2, per 1% increase	0.026 (0.001–0.841)	0.04
Respiratory rate, breaths/min	0.996 (0.992–1.000)	0.045
spo2_fi_o2_mm_hg	1.086 (1.001–1.178)	0.048
ast_u_l	1.000 (1.000–1.000)	0.075
White blood cells, ×10^9/L	1.072 (0.990–1.160)	0.086
sbp_mm_hg	0.985 (0.968–1.003)	0.1
icu_stays	4.575 (0.614–34.065)	0.138
pa_o2_fi_o2_mm_hg	0.997 (0.993–1.001)	0.153
temperature_c	1.199 (0.931–1.544)	0.16
alt_u_l	1.000 (1.000–1.000)	0.172
t_bil_umol_l	1.002 (0.998–1.007)	0.255
gcs	0.947 (0.860–1.043)	0.268
pa_o2_mm_hg	0.989 (0.969–1.010)	0.304
fi_o2	1.291 (0.456–3.657)	0.631

Footnote: HR = hazard ratio; CI = confidence interval. Univariable Cox proportional hazards models were fitted for each candidate covariate. P values <0.001 are reported as <0.001.

Univariable Cox proportional hazards models were constructed for all candidate clinical variables to evaluate their associations with the risk of all-cause in-hospital mortality. Results are presented as hazard ratios (HRs) with 95% confidence intervals (CIs). Continuous variables were entered into the models using their original scales or prespecified clinically interpretable units (e.g., serum creatinine per 10 μmol/L increase and platelet count per 10×10^9/L increase). Binary variables were coded as 0/1. P values <0.001 were uniformly reported as P<0.001.

The univariable analyses showed that multiple indicators reflecting disease severity, organ dysfunction, and supportive treatment requirements were significantly associated with in-hospital mortality risk. Among scoring systems, HSSS was positively associated with mortality (HR=1.222, 95% CI 1.112–1.342, P<0.001). Variables related to organ support and critical care management, including renal replacement therapy (RRT), vasopressor use, and acute gastrointestinal injury (AGI) grade, demonstrated strong associations with increased mortality risk (all P<0.001).

Among vital signs and laboratory parameters, higher heart rate was associated with increased mortality risk (HR=1.036, 95% CI 1.018–1.055, P<0.001), whereas higher mean

arterial pressure, oxygen saturation, and platelet count were associated with lower mortality risk. Renal function indicators also showed statistically significant associations; for example, higher serum creatinine levels (per 10 μmol/L increase) were associated with increased in-hospital mortality risk (HR=1.030, 95% CI 1.008–1.053, P=0.009).

Importantly, univariable Cox analyses were not adjusted for potential confounders; therefore, these results represent unadjusted associations and should not be interpreted causally. Accordingly, this table is primarily intended to describe preliminary risk characteristics of candidate variables and, in conjunction with clinical plausibility, interpretability, and inter-variable correlations, to support covariate selection and model construction for subsequent multivariable Fine–Gray regression models and conventional multivariable Cox regression analyses.

Table.S3 Univariable logistic regression analyses of associations between clinical scoring systems and prolonged hospitalization among survivors

outcome	score	OR	lo	hi	p	p_label
**prolonged_stay_p75**	SOFA1	1.1847	1.0681	1.3226	1.72E-03	0.002
**prolonged_stay_p75**	SOFA2	1.1735	1.0677	1.2980	1.21E-03	0.001
**prolonged_stay_p75**	MEWS	1.3512	1.1479	1.6129	4.76E-04	<0.001
**prolonged_stay_p75**	NEWS	1.2280	1.0945	1.3896	6.98E-04	<0.001
**prolonged_stay_p75**	HSSS	1.2181	1.0923	1.3712	6.00E-04	<0.001
**extremely_prolonged_stay_p90**	SOFA1	1.2156	1.0609	1.3981	4.90E-03	0.005
**extremely_prolonged_stay_p90**	SOFA2	1.2143	1.0734	1.3831	2.28E-03	0.002
**extremely_prolonged_stay_p90**	MEWS	1.0897	0.8700	1.3525	4.41E-01	0.441
**extremely_prolonged_stay_p90**	NEWS	1.1062	0.9462	1.2999	2.09E-01	0.209
**extremely_prolonged_stay_p90**	HSSS	1.1963	1.0429	1.3729	9.27E-03	0.009

OR = odds ratio；CI = confidence interval.
Univariable logistic regression models were fitted for each clinical scoring system.
Prolonged hospitalization was defined as length of hospital stay ≥ 75th percentile (P75) among survivors, and extremely prolonged hospitalization as length of stay ≥ 90th percentile (P90).
Analyses were restricted to patients who survived to hospital discharge.

This table presents results from univariable logistic regression analyses evaluating the associations between different clinical scoring systems (SOFA1, SOFA2, MEWS, NEWS, and HSSS) and prolonged length-of-stay outcomes among patients discharged alive. Prolonged hospitalization was defined as a length of stay greater than or equal to the 75th percentile (P75) of the distribution among survivors, whereas extremely prolonged hospitalization was defined as a length of stay greater than or equal to the 90th percentile (P90). All analyses were restricted to patients who survived to discharge, and in-hospital deaths were excluded.

Logistic regression results are reported as odds ratios (ORs) with 95% confidence intervals (CIs). ORs represent the relative change in the odds of the corresponding prolonged hospitalization outcome per 1-point increase in the score.

For the P75 prolonged hospitalization outcome, all included scoring systems (SOFA1, SOFA2, MEWS, NEWS, and HSSS) showed significant positive associations with prolonged length of stay, suggesting that survivors with greater admission severity were more likely to experience prolonged hospitalization. For the P90 extremely prolonged hospitalization outcome, associations differed across scoring systems. SOFA1, SOFA2, and HSSS remained significantly associated with extremely prolonged hospitalization, whereas MEWS and NEWS were not statistically significant. These findings suggest potential differences in the ability of different scoring systems to discriminate more extreme hospitalization burden among survivors.

All results shown in this table are derived from univariable analyses and are intended to describe preliminary association patterns between scoring systems and length-of-stay outcomes, serving as supportive information for interpretation of subsequent findings.

Table S4. Approximate events-per-variable (EPV), effective degrees of freedom, and interpretive considerations for the main regression and subtype-specific analyses

Analysis component	Cohort used	Death events	Parameters / effective df	Approx. EPV	Comment
Cause-specific Cox, linear SOFA1 + age	Overall cohort	24	2	12	Minimal adjusted model
Cause-specific Cox, linear SOFA2 + age	Overall cohort	24	2	12	Minimal adjusted model
Cause-specific Cox, RCS SOFA1 (3 knots) + age	Overall cohort	24	3	8	SOFA1 contributed 2 df; age 1 df
Cause-specific Cox, RCS SOFA2 (3 knots) + age	Overall cohort	24	3	8	SOFA2 contributed 2 df; age 1 df
Fine-Gray, linear score + age*	Overall cohort	24	2	12	If fitted as score + age
Fine-Gray, RCS score (3 knots) + age	Overall cohort	24	3	8	Same df structure as above
Subtype 0 descriptive ROC/DCA	Subtype 0	3	—	—	Extremely underpowered; inferential interpretation should be avoided
Subtype 1 descriptive ROC/DCA	Subtype 1	21	—	—	More informative than subtype 0, but still limited
Gaussian mixture clustering	Overall cohort	24 deaths overall	—	—	EPV is not applicable; the concern lies in the scarcity of events relative to downstream interpretation, rather than regression overfitting itself.

Abbreviations: EPV, events per variable; df, degrees of freedom; RCS, restricted cubic spline; DCA, decision curve analysis; ROC, receiver operating characteristic. Approximate EPV was calculated as the number of in-hospital death events divided by the number of model parameters or effective degrees of freedom. In RCS models with 3 knots, the score term contributed 2 effective df and age contributed 1 df. EPV is not directly applicable to descriptive ROC/DCA analyses or Gaussian mixture clustering. This table is intended to contextualize model complexity and the limited statistical power of subtype-specific analyses.

Supplementary Table S4 is intended to provide a transparent overview of event burden relative to analytic complexity across the main models used in this study. In the overall cohort, the minimally adjusted time-to-event models were relatively parsimonious, with approximately 12 events per variable for linear score + age models and approximately 8 events per effective degree of freedom for restricted cubic spline models. However, after stratification by clinical subtype, the number of death events became markedly imbalanced, with only 3 deaths in Subtype 0 and 21 deaths in Subtype 1. Accordingly, subtype-specific ROC and decision curve analyses should be interpreted primarily as descriptive and exploratory, especially in Subtype 0, where inferential interpretation would be highly unstable.

For unsupervised Gaussian mixture clustering, conventional EPV is not directly applicable because clustering itself is not a regression model for mortality. Instead, the interpretive concern lies in the limited number of downstream death events available for evaluating subtype-specific prognostic patterns. Overall, these results support cautious interpretation of subgroup findings and reinforce that subtype-level analyses in the present study should be viewed as hypothesis-generating rather than definitive.

3 Supplementary Figures(S1 to S6)

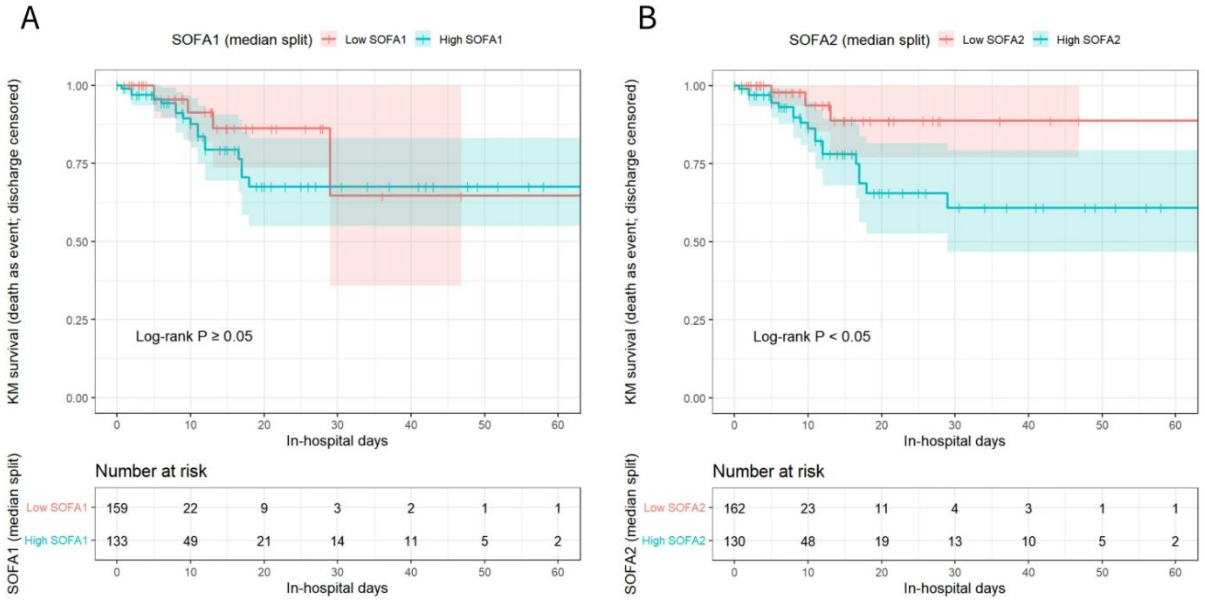

Fig S1. Kaplan–Meier survival analysis stratified by median split of SOFA1 and SOFA2.

Kaplan–Meier survival analyses were performed to descriptively assess the associations between baseline SOFA1 and SOFA2 scores and in-hospital survival based on all-cause in-hospital mortality. SOFA1 and SOFA2 were each dichotomized at their respective medians in the study cohort, classifying patients into low-score (Low) and high-score (High) groups. Survival differences between groups were compared using the log-rank test.

(A) Kaplan–Meier survival curves stratified by median-dichotomized SOFA1.

Compared with the low SOFA1 group, patients in the high SOFA1 group showed an overall trend toward lower survival probability during hospitalization; however, substantial overlap was observed between the survival curves, and the between-group difference did not reach statistical significance (log-rank $P > 0.05$). These results suggest that SOFA1 may have limited discriminative ability for in-hospital mortality risk under a median-dichotomization strategy.

(B) Kaplan–Meier survival curves stratified by median-dichotomized SOFA2.

Patients in the high SOFA2 group exhibited significantly lower in-hospital survival probability than those in the low SOFA2 group, with a statistically significant difference between groups (log-rank $P < 0.05$). Compared with SOFA1, SOFA2 demonstrated clearer risk stratification under the same grouping strategy.

For both panels, the number at risk is displayed at each time point, and shaded areas represent 95% confidence intervals for the survival function. Notably, this Kaplan–Meier analysis treated discharge alive as a censoring event and did not account for competing risks; therefore, it is presented only as a supplementary and intuitive visualization complementing the competing-risk models and continuous-variable analyses reported in the main text.

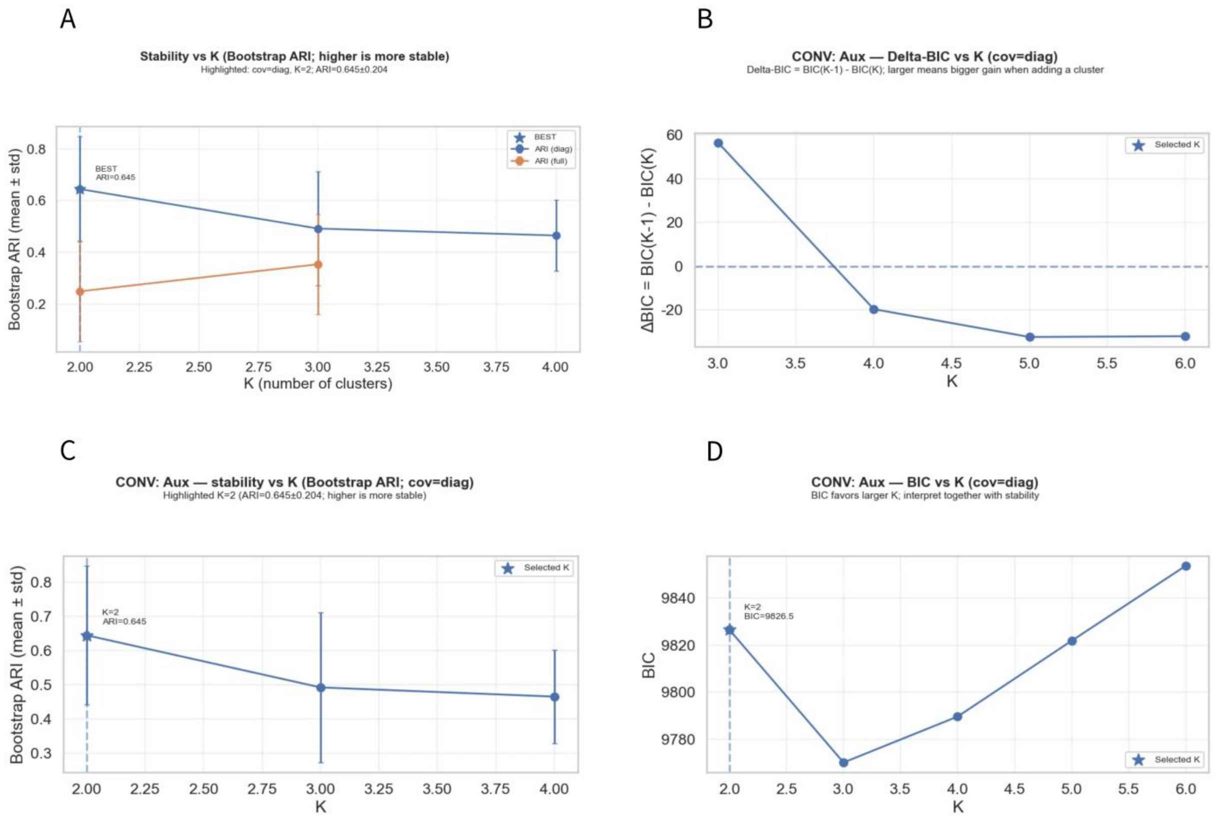

Fig S2. Evaluation of clustering stability and model fit across different numbers of clusters (K)

Gaussian mixture models (GMMs) were evaluated across candidate cluster numbers (K = 2–6). The final number of clusters was selected by jointly considering clustering stability and model goodness-of-fit.

(A) Bootstrap-based clustering stability analysis. Adjusted Rand index (ARI) values (mean ± SD) are shown for diagonal (diag) and full covariance structures. Diagonal covariance models demonstrated consistently higher stability, with the highest mean ARI observed at K = 2.

(B) ΔBIC across K. ΔBIC was calculated as BIC(K−1) − BIC(K) to quantify marginal improvement in model fit when increasing K. The largest ΔBIC was observed at K = 3 (corresponding to the transition from K = 2 to K = 3), whereas further increases in K provided diminishing or negative marginal gains.

(C) Stability under diagonal covariance structure. Under the diagonal covariance model, bootstrap-derived ARI was highest at K = 2 and gradually decreased with larger K values.

(D) Bayesian information criterion (BIC) across K. The lowest BIC was observed at K = 3 under the diagonal covariance model. However, when stability metrics and model parsimony were considered jointly, K = 2 provided a more robust and reproducible clustering solution for the present dataset.

Collectively, these results support K = 2 as the primary phenotyping solution.

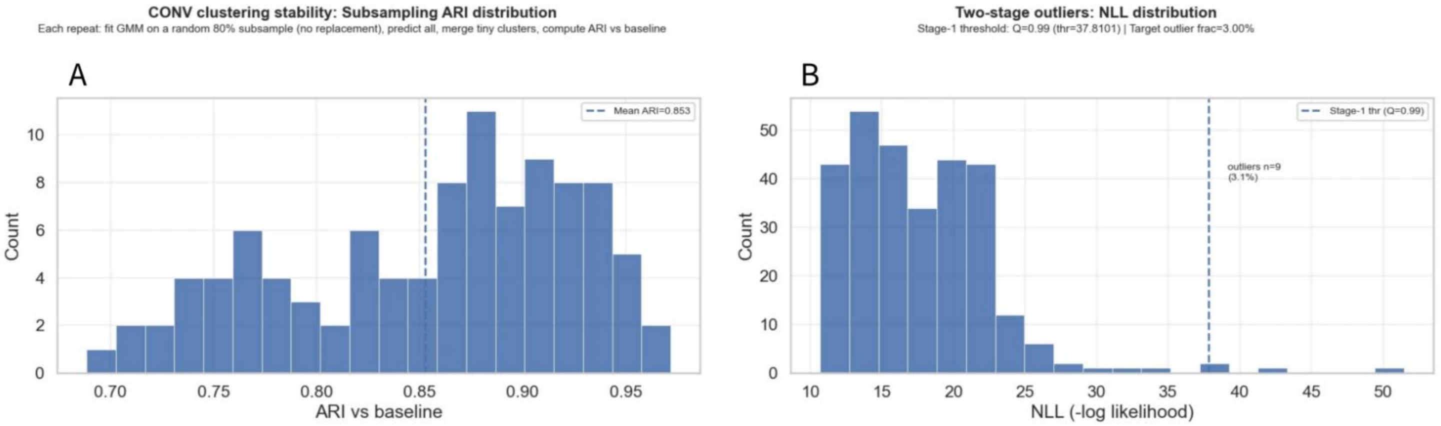

Fig S3. Stability assessment and outlier identification for the final clustering solution.

To further evaluate the robustness of the final clustering solution (K=2) and to assess the potential influence of extreme individuals on the phenotyping structure, subsampling-based stability analyses and outlier identification procedures were performed.

(A) Subsampling-based stability assessment. In each iteration, 80% of the cohort was randomly sampled without replacement, and the Gaussian mixture model was refitted. Cluster labels derived from each subsampled model were compared with the baseline phenotyping solution (100 repetitions), and clustering agreement was quantified using the adjusted Rand index (ARI). The histogram displays the distribution of ARI values across repetitions, and the dashed line indicates the mean ARI. Results showed that ARI values remained high in most repetitions, suggesting good reproducibility and stability of the final phenotyping solution.

(B) Two-stage outlier identification based on negative log-likelihood (NLL). Potential outlier individuals were first screened using the 99th percentile threshold of NLL values in the full cohort. The outlier proportion was then further controlled, and a total of 9 patients (approximately 3.1% of the cohort) were ultimately identified as rare/outlier samples. These individuals exhibited probability densities markedly deviating from the main distribution and were therefore excluded from the construction of the primary subtypes and labeled as a separate rare/outlier group.

Collectively, these analyses indicate that the final clustering solution demonstrated high stability across repeated subsampling, and that the main subtype structure was not driven by a small number of extreme samples.

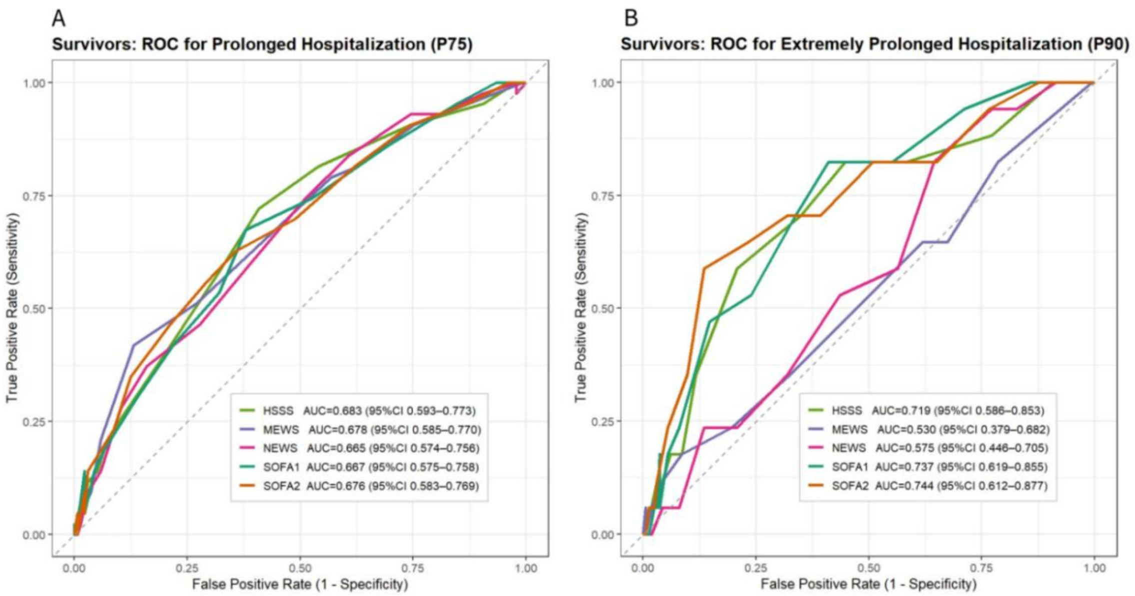

Fig S4. Receiver operating characteristic (ROC) analyses of clinical scoring systems for prolonged hospitalization among survivors

To further evaluate the discriminative ability of baseline admission severity scores for hospitalization burden–related outcomes among survivors, receiver operating characteristic (ROC) curve analyses were performed for prolonged hospitalization (P75) and extremely prolonged hospitalization (P90) in patients discharged alive.

(A) ROC curve analysis for prolonged hospitalization (P75). Prolonged hospitalization was defined as a length of stay greater than or equal to the 75th percentile (P75) of the distribution among survivors. Discriminative performance across SOFA1, SOFA2, MEWS, NEWS, and HSSS was modest overall, with broadly comparable AUC estimates, suggesting limited ability of any single admission score to distinguish prolonged hospitalization among survivors.

(B) ROC curve analysis for extremely prolonged hospitalization (P90). Extremely prolonged hospitalization was defined as a length of stay greater than or equal to the 90th percentile (P90) of the distribution among survivors. Under this more extreme outcome threshold, numerical differences in AUC estimates became somewhat more apparent; however, overall discriminative performance remained moderate and these differences should be interpreted cautiously.

Overall, these ROC analyses suggest that baseline admission severity scores may capture some degree of hospitalization burden among survivors, but their discrimination for length-of-stay–related outcomes was limited. These secondary analyses are presented as supplementary and exploratory observations and should not be interpreted as evidence of superiority of any individual scoring system.

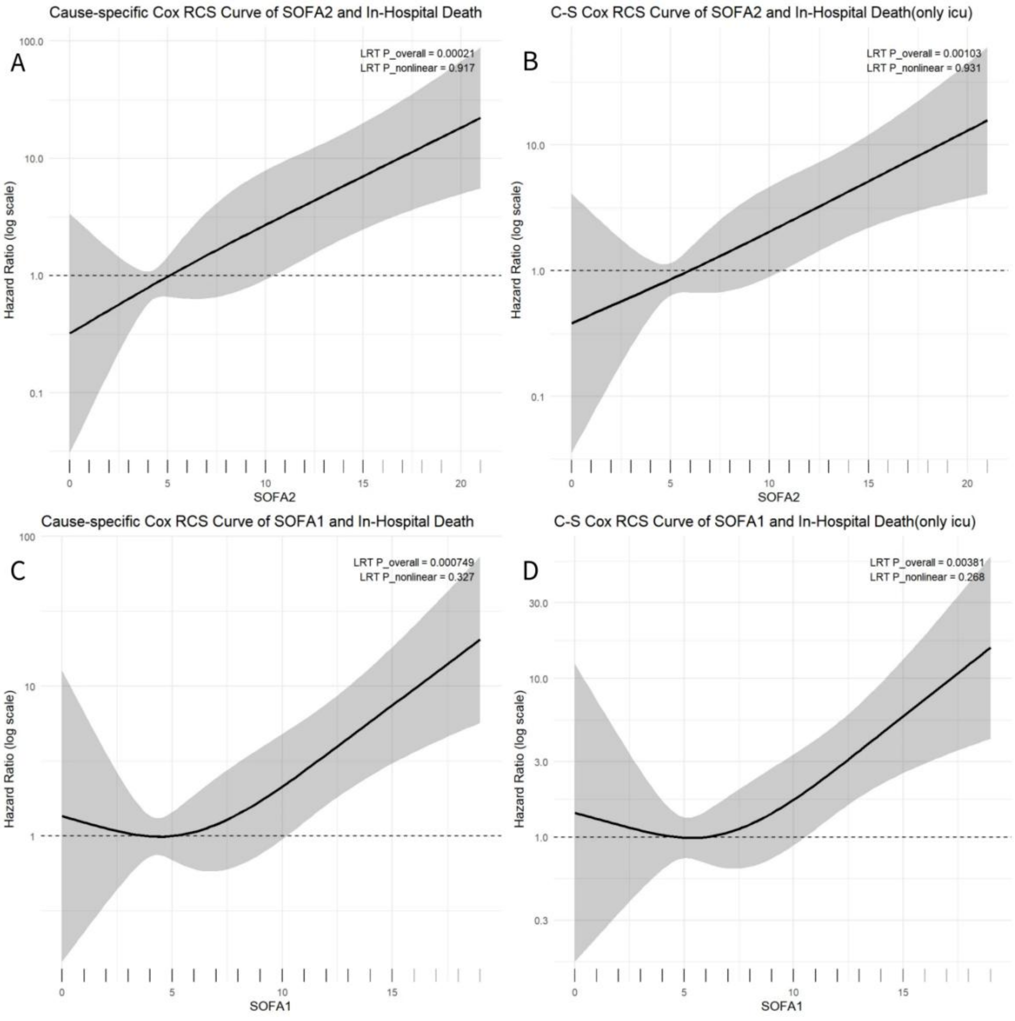

Fig S5. Restricted cubic spline (RCS) analyses of the associations between SOFA2 and SOFA1 scores and in-hospital mortality using cause-specific Cox proportional hazards models

To assess the robustness of the primary competing-risk findings under a conventional proportional hazards framework, restricted cubic spline (RCS) analyses were further performed using cause-specific Cox proportional hazards models to evaluate the associations of SOFA2 and SOFA1 scores with in-hospital mortality. Models were constructed with minimal adjustment for age, and the proportional hazards assumption was examined using Schoenfeld residual tests (see Supplementary Figure).

(A) RCS curve for SOFA2 in the overall cohort.

(B) RCS curve for SOFA2 in the ICU subgroup.

(C) RCS curve for SOFA1 in the overall cohort.

(D) RCS curve for SOFA1 in the ICU subgroup.

The x-axis represents SOFA scores and the y-axis represents hazard ratios (HRs; log scale), using the median score as the reference (HR = 1). Solid lines indicate estimated risk trends,

shaded areas represent 95% confidence intervals, and dashed lines denote the reference level. P_overall and P_nonlinear were derived from likelihood ratio tests (LRT) to evaluate the overall association and the nonlinear component, respectively. Results showed that higher SOFA1 and SOFA2 scores were associated with increased in-hospital mortality risk in both the overall cohort and the ICU subgroup, with no statistically significant evidence of nonlinearity.Results showed that higher SOFA1 and SOFA2 scores were associated with increased in-hospital mortality risk in both the overall cohort and the ICU subgroup, with no statistically significant evidence of nonlinearity. For both scores, the observed associations were directionally consistent with an approximately monotonic positive trend across the score range. Overall, these patterns were consistent with the competing-risk regression findings reported in the main analyses.

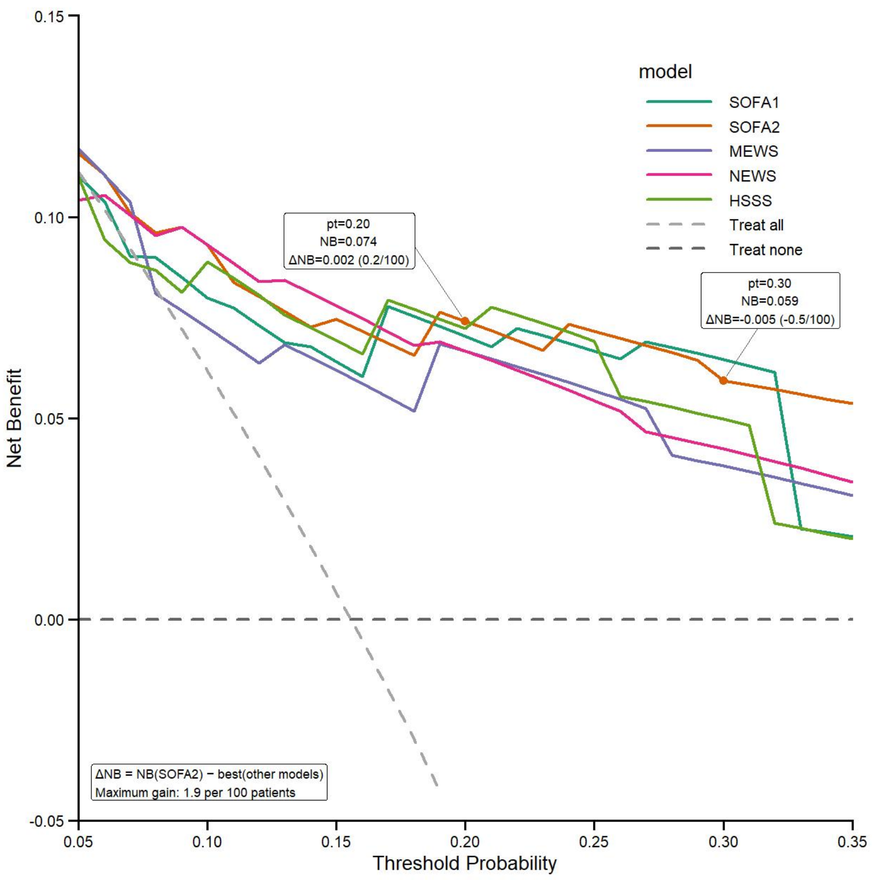

Fig S6. Decision curve analysis of clinical net benefit of different scoring systems in Subtype 1

Decision curve analysis (DCA) was performed to descriptively compare the potential clinical decision value of different baseline admission scoring systems (SOFA1, SOFA2, MEWS, NEWS, and HSSS) for predicting in-hospital mortality risk in Subtype 1. The x-axis represents threshold probability and the y-axis represents net benefit. The dashed lines indicate the reference strategies of “treat all” and “treat none.”

Across the evaluated threshold range, several scoring systems showed positive net benefit relative to the default strategies. Numerical differences between models were observed within certain intervals, but given the limited number of events and the exploratory nature of this analysis, these patterns should be interpreted cautiously and descriptively rather than inferentially.

ΔNB indicates the difference in net benefit between SOFA2 and the best-performing comparator at a given threshold, expressed as the incremental number of net correct decisions

per 100 patients.

As noted in the main text, because death events were sparse in Subtype 0, DCA estimates in that subtype were unstable and were not emphasized. This figure is therefore presented as a supplementary exploratory analysis in Subtype 1.
